# Bonds improve post mission disposal compliance and the sustainability of low Earth orbit

**DOI:** 10.1038/s41467-026-77660-4

**Published:** 2026-09-15

**Authors:** I. Brownhall, D. Kaffine, D. Kilpatrick, M. Lifson, M. Moretto, E. Stevenson, S. Lemmens, F. Letizia, S. Bhattarai

**Affiliations:** 1 University College London (UCL), OrbitLab, Department of Engineering, London, UK; 2 University of Colorado Boulder, Department of Economics, Renewable and Sustainable Energy Institute, Boulder, Colorado USA; 3 University of Colorado Boulder, Department of Economics, Boulder, Colorado USA; 4 The Aerospace Corporation, Virginia, USA; 5 North Carolina State University, Department of Mechanical and Aerospace Engineering, Raleigh, USA; 6 IMS Space Consultancy at ESA ESTEC, Noordwijk, The Netherlands; 7 European Space Agency, ESTEC, Noordwijk, The Netherlands

**Keywords:** Space physics, Sustainability, Economics

## Abstract

Post-mission disposal (PMD) non-compliance threatens the long-term sustainability of Low Earth Orbit (LEO). Between 2010 and 2024, only 40% of satellites in non-naturally compliant orbits successfully deorbited. As launch rates and large constellations expand the active population, reducing satellite derelicts is increasingly urgent. We evaluate an escrow-based PMD performance bond that makes non-compliance financially costly while preserving access to orbit. The analysis couples an economic model of operator decision-making with a model of LEO population evolution, allowing launches and debris risk to co-evolve over 25 years. Bonds of $200,000 per satellite reduce derelict satellite mass per year by more than one-third and increase welfare by up to 27%, while bonds above $1 million show diminishing marginal returns. Even partial adoption can be effective: a bond covering 20% of the global market still improves sustainability under all levels of market leakage, although broader participation delivers greater environmental and economic benefits.

## Abstract (web summary)

Growing satellite populations and unreliable operational behaviour could lead to an increase in orbital debris. This study finds that refundable performance bonds can encourage safer disposal, reduce long-term space debris and improve economic welfare, even when only part of the market adopts them.

## Introduction

For decades, the mass of space debris in orbit has exceeded the number of operational satellites^1^. In recent years, space activity has accelerated sharply: launch rates are now roughly 20 times higher than two decades ago, and by 2030 the number of active satellites could reach 100,000 to 1 million, driven by large Low Earth Orbit (LEO) constellations^2^. Estimates suggest there are already around 1.5 million debris objects larger than 1 cm, and under modest extrapolations of current trends, by 2225, there could be approximately 750–800 cumulative collisions and roughly 150,000 trackable objects larger than 10 cm^3^. This growing congestion threatens long-term access to orbit and undermines satellite services, including those that support the United Nations’ Sustainable Development Goals (SDGs)^4^. LEO is therefore best understood as a common-pool resource system with limited capacity, where misaligned incentives and open access are generating a “tragedy of the commons”^5^. Of particular concern is failure to remove satellites from orbit after their active lifetime - post mission disposal (PMD)—as these derelicts can lead to fragmentation and increased collision risk. Inter-Agency Space Debris Coordination Committee (IADC) guidelines suggest deorbiting within 25 years, but some national regulators have implemented stricter 5-year PMD limits for their registered satellites^6^. Nevertheless, compliance rates in LEO between 2010–2024 remain well below the IADC’s 90% compliance recommendation: manoeuvrable compliant satellites having average compliance rates of just 45%^3^. Globally, national space regulatory agencies are actively exploring new policies to address this growing sustainability issue^7–9^, given the risks posed to satellite services such as telecommunications, earth observation, and positioning and navigation.

Low levels of PMD compliance, partly because existing PMD guidelines are voluntary and not legally binding, pose a risk to the long-run sustainability and safety of LEO^10^. While widespread PMD would reduce long-term debris growth, there remains a key knowledge gap regarding which policy designs can reliably raise PMD compliance without unduly suppressing beneficial space activity. Insights from environmental economics may help fill this gap. In sectors such as climate^11^, marine resources^12^, waste^13^, and air pollution^14^, fiscal instruments are used to better align private incentives with socially efficient outcomes. Increasing regulatory compliance through financial bonds has been widely used in Earth-based resource management^15,16^, and bonds for PMD have been proposed for the space environment^17–19^. However, despite growing interest in PMD performance bonds, their effects on both space sustainability and economic welfare have not yet been quantitatively evaluated using an integrated assessment model (IAM) that links operator incentives, market responses and the evolving debris environment across multiple satellite types. This is an important gap because policy-induced changes in prices, demand, and substitution can alter launch activity and disposal behaviour, shaping the net environmental outcome.

**Fig. 1:**
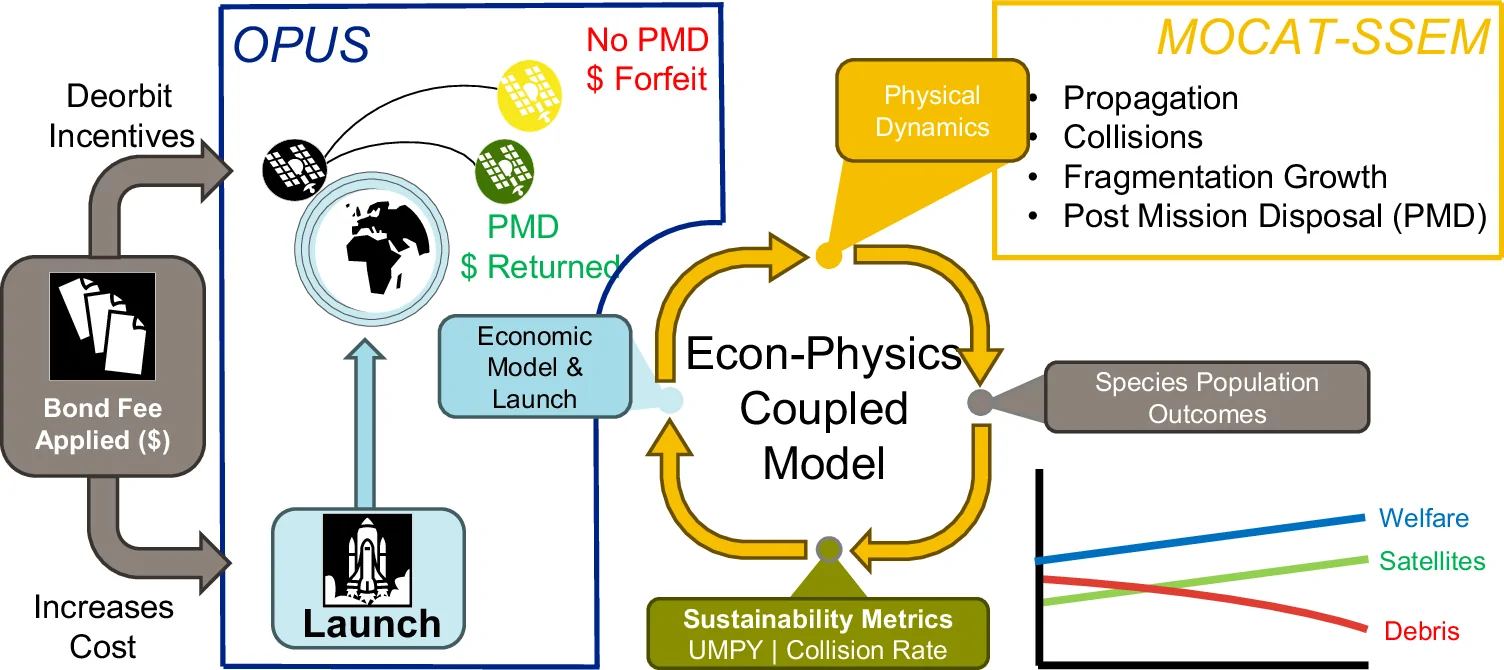
A visual description of the Orbital Debris Propagators Unified with Economic Systems (OPUS) integrated assessment model, as implemented by the authors. OPUS is the economic module, which is coupled with the physical debris model, MIT Orbital Capacity Assessment Tool- Source Sink Evolutionary Model (MOCAT-SSEM)^34^. The launch model is endogenously handled by OPUS, with launch decisions passed to MOCAT-SSEM, which are then propagated forward one year. Species populations plus key sustainability metrics are passed back, which informs future launch decisions. This repeats for the simulation length, 25 years. UMPY is the Undisposed Mass Per Year and is a measure of space sustainability^43^. Operators pay a bond when launching satellites, which is returned with interest if they successfully complete the required post-mission disposal.

To address this gap, this paper quantitatively assesses the impact of a PMD bond designed to discourage post-mission abandonment of non-naturally compliant derelicts (Fig. 1). We propose an escrow-based mechanism, administered by a third party (e.g., a government), in which a fee imposed on satellite operators is collected by the launching state and invested at market interest rates. If the operator successfully completes PMD, the full contribution is returned with interest. Otherwise, the funds are retained by the regulator, and in theory, the revenues could be re-invested to support initiatives such as debris removal^20^. In other words, satellite operators pay a deposit when they put a satellite in orbit, and receive a refund (with interest) if they successfully deorbit. To analyse the bond’s efficacy, we use the Orbital Debris Propagators Unified with Economic Systems (OPUS) Integrated Assessment Model (IAM). This integrated framework captures how a bond influences operator behaviour, and through resulting launch and disposal decisions, the long-term debris environment and the sustainability of space (see Fig. 1 and “Methods”). We conduct 25-year simulations across bond levels up to $2 million per satellite. For varying bond levels, we evaluate outcomes under both 5-year and 25-year PMD deorbit rules.

We answer three research questions. First, what is the impact of a PMD bond on space sustainability metrics and welfare if all satellites are subject to the bond? Second, how do the effects vary if the bond is returned upon reaching a compliant indirect disposal orbit versus direct disposal? And third, in a global market context with heterogeneous PMD regulatory practice and market leakage, how would a PMD bond policy perform if only applied in a small market?

Overall, we find that bonds improve space sustainability and increase economic welfare by reducing debris and risk. Notably, even modest bond amounts can improve space sustainability under both 5- and 25-year PMD lifetimes. Sustainability and welfare outcomes are sensitive to the requirements for bond refunds, with bond refunds for direct disposal proving to be more effective at improving sustainability than only requiring indirect disposal. Finally, we find that bonds applied to only a subset of satellites can still improve sustainability and economic welfare, with effectiveness depending on rates of market substitution. In general, these results provide quantitative evidence for PMD bonds as a promising regulatory tool for improving space sustainability.

## Results

### Disposal bonds can reduce debris and increase economic welfare

**Fig. 2:**
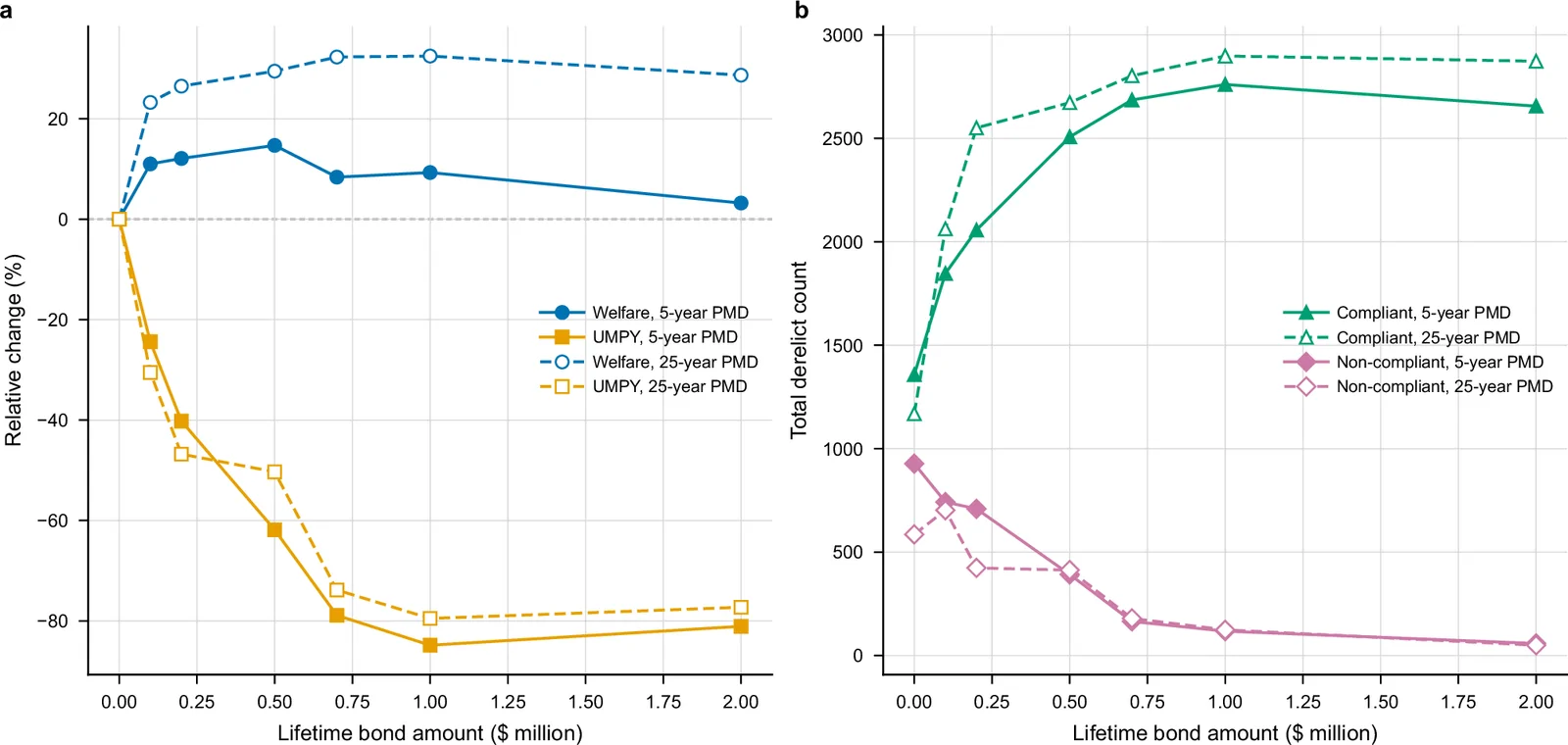
Effects of lifetime bond value on welfare, debris burden and derelict satellites. **a** A relative changes in final-year welfare and Undisposed Mass Per Year (UMPY) compared with the no-bond baseline. UMPY measures the environmental burden associated with uncontrolled objects and their orbital persistence, as defined in the “Methods”. **b** Total numbers of compliant and non-compliant derelict satellites, as a function of bond value. Operators are classified as compliant when their satellites are placed in orbits that decay within the applicable 5- or 25-year post-mission disposal (PMD) limit. For context, bonds of $100,000 and $500,000 correspond to approximately 3.5% and 17%, respectively, of the average total satellite cost. Solid lines and filled markers represent the 5-year PMD case; dashed lines and open markers represent the 25-year PMD case. Blue circles and orange squares denote welfare and UMPY, respectively; turquoise triangles and magenta diamonds denote compliant and non-compliant derelicts, respectively.

Figure 2 presents that as the PMD bond fee increases, there is a substantial reduction in non-compliant derelict satellites, alongside an increase in compliant derelicts that are placed on orbits meeting the 5-year or 25-year disposal rules. This shift in the derelict population explains the reduction in Undisposed Mass Per Year (UMPY, see “Methods” for definition) over the 25-year simulation horizon, as derelicts are not left on orbit after their active lifetime. This implies that a well-designed PMD bond policy strengthens PMD compliance, reducing the number of derelicts and improving space sustainability. Across the bond levels considered, economic welfare (see “Methods” for calculation details) is above the baseline, indicating that sustainability gains are Pareto-improving rather than coming at an economic cost.

Under a 5-year PMD rule, a $100k bond (3.5% of the average satellite cost) reduces UMPY by 24% relative to the baseline. In comparison, for a 25-year rule, there is a reduction in UMPY of 31%. Raising the bond to $500 k (17% of the average satellite cost) yields larger improvements in UMPY (~50–65% reductions), though beyond $0.75 M, reductions in the UMPY plateau at an ~80% reduction. At these larger bond amounts with high compliance rates, bonds are modestly more effective at reducing UMPY under a 5-year PMD rule. Under a 25-year PMD rule, derelict satellites remain at higher altitudes for longer along elliptical disposal orbits (see “Methods”) and traverse more operational shells, thereby raising collision risk and debris accumulation. Non-compliant bond revenues are maximised at a $500 k bond price (see SI Table S4), collecting $2.75B under a 5-year deorbit rule and $3.05B under a 25-year deorbit rule, and decreasing to $1.87B and $ 1.91B for a $2 million bond under 5- and 25-year deorbit rules, respectively. Finally, the bond increases economic welfare relative to the baseline for all bond amounts. However, on the margin, welfare gains begin to flatten around $500 k and then decline for the largest bond amounts, as the launch disincentives from the bond (and corresponding compliance costs) begin to outweigh the diminishing benefits from reductions in collision risk.

These results indicate that a PMD bond is an effective instrument for improving the sustainability of LEO via a sharp reduction in new non-compliant derelicts across all satellite classes (see detailed breakdown in SI4). Importantly, most of the environmental benefit is realised at relatively modest bond levels: under a 5-year PMD deorbit rule, bonds in the $100–500 k range deliver large reductions in derelicts and UMPY while maintaining a substantial active satellite population and improved welfare. Higher bond amounts continue to improve the environment but offer smaller incremental gains and begin to erode the number of active satellites and welfare on the margin.

From a policy perspective, this suggests that PMD bonds can internalise the external costs of debris without severely constraining economic activity in LEO. The existence of a broad range of bond values that both lower UMPY and increase the active satellite population provides flexibility for regulators to balance or prioritise environmental and economic benefits.

### Below a $500 k bond, requiring direct disposal for bond return is more effective at reducing UMPY

**Fig. 3:**
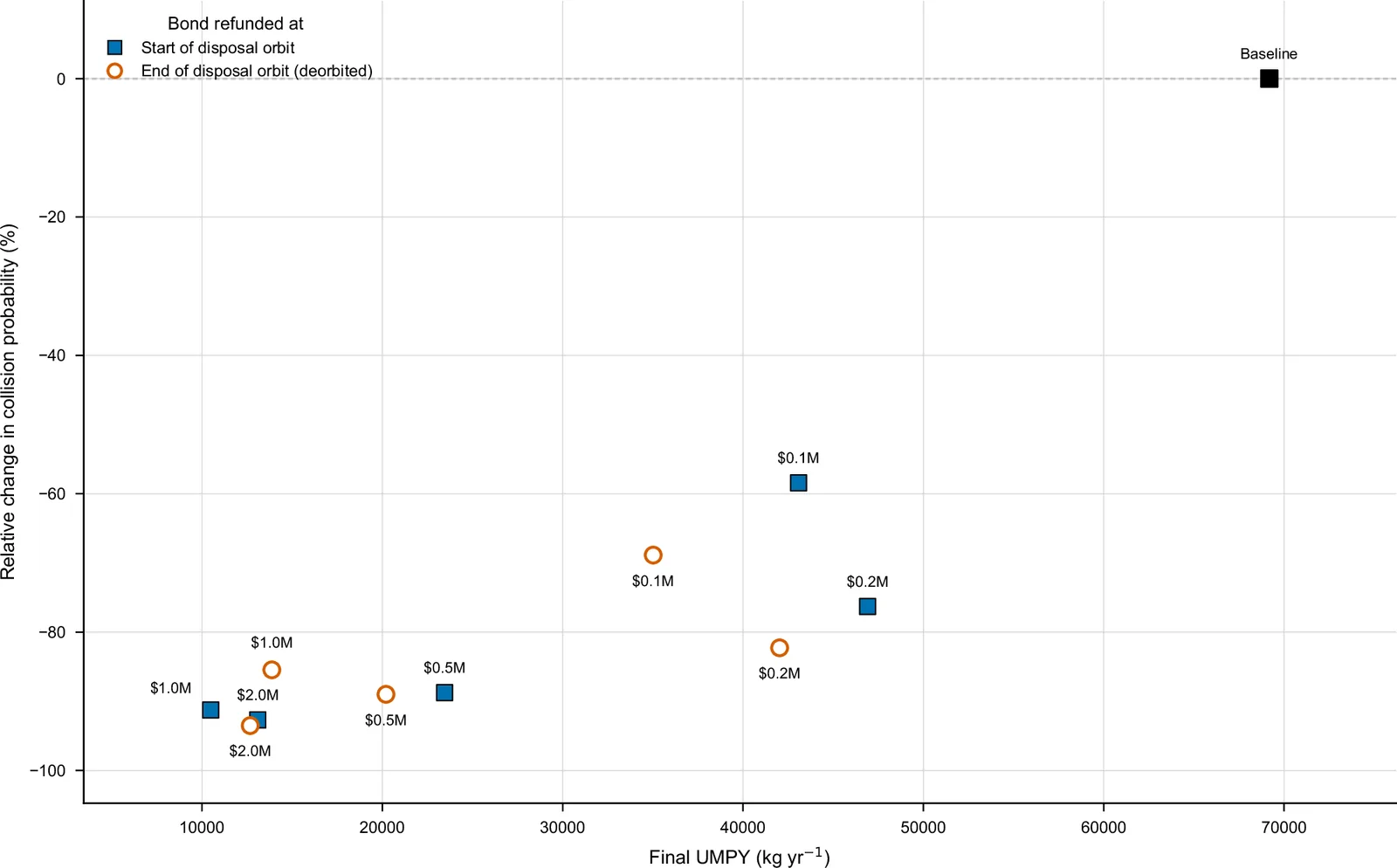
Relationship between final UMPY and collision probability under a 5-year deorbit rule. Final-year Undisposed Mass Per Year (UMPY) is plotted against the relative change in collision probability compared with the indirect post-mission disposal (PMD), no-bond baseline. Each labelled point represents a different bond value. Squares denote bonds refunded when the satellite enters a compliant disposal orbit, whereas circles denote bonds refunded only after deorbiting. Lower values on both axes indicate improved environmental outcomes.

Figure 3 presents the final UMPY achieved for a given bond level under a 5-year deorbit rule, together with the corresponding relative change in collision probability compared to the baseline. Requiring powered direct disposal (orange circle) for a bond refund generally leads to greater reductions in UMPY and collision risks than only requiring a satellite to be placed into a compliant indirect disposal orbit (blue square) (see “Methods” for a detailed description of the disposal orbit types). However, for bond fees below $1 million, requiring direct disposal reduces economic welfare relative to indirect disposal; at higher bond fees, a cleaner overall environment encourages launch and improves welfare (see SI Fig. S9).

This distinction in the requirements and conditions for disposal bond return is an important policy design choice, as the bond policy introduces a classic intensive versus extensive margin trade-off. Space sustainability can be improved if operators who comply with PMD use cleaner disposal methods, such as direct disposal (the intensive margin). Alternatively, space sustainability can also be improved if more operators comply with PMD in the first place, regardless of disposal method, or indirect disposal (the extensive margin). The choice of requirements and conditions for bond return creates a meaningful behavioural difference for operators, as a direct disposal requires substantially more fuel, raising the opportunity cost of complying with PMD and making it more attractive to continue operating in orbit (and forfeiting the bond). As such, targeting the intensive margin by requiring cleaner direct disposal weakens the extensive margin as fewer operators could comply. These countervailing effects mean that theory alone cannot answer which approach best enhances space sustainability.

Introducing a bond reduces UMPY considerably in both scenarios, but the effects diverge the most at low bond levels. At bond amounts of $100–200 k, the benefits of cleaner direct disposal (intensive margin) outweigh less compliance (extensive margin), resulting in lower UMPY and larger reductions in collision probability when bond refund requires direct disposal. In other words, at these lower bond amounts, even though the marginal financial benefit of recovering the bond is insufficient to offset the high propellant cost of a direct deorbit for some operators (forgoing PMD), the cleaner disposal method for those who do comply generates greater improvements in space sustainability. Once the bond exceeds approximately $500–750 k, the gap between the two scenarios narrows markedly. At these levels, the financial incentive generally outweighs the opportunity cost of direct disposal, leading operators in both scenarios to comply at similar high rates. As a result, UMPY continues to fall, and collision probability converges between the two disposal criteria.

The refund requirements affect UMPY and collision in distinct ways that may be relevant for policy. Under indirect disposal, UMPY is lowered via the movement of derelicts into disposal orbits, where they eventually decay. By contrast, with direct disposal, the removal of mass is immediate and complete by the (relatively fewer) operators who comply, producing sharper improvements in collision risk on net for a given UMPY level.

### Even a small market can impose an effective bond

**Fig. 4:**
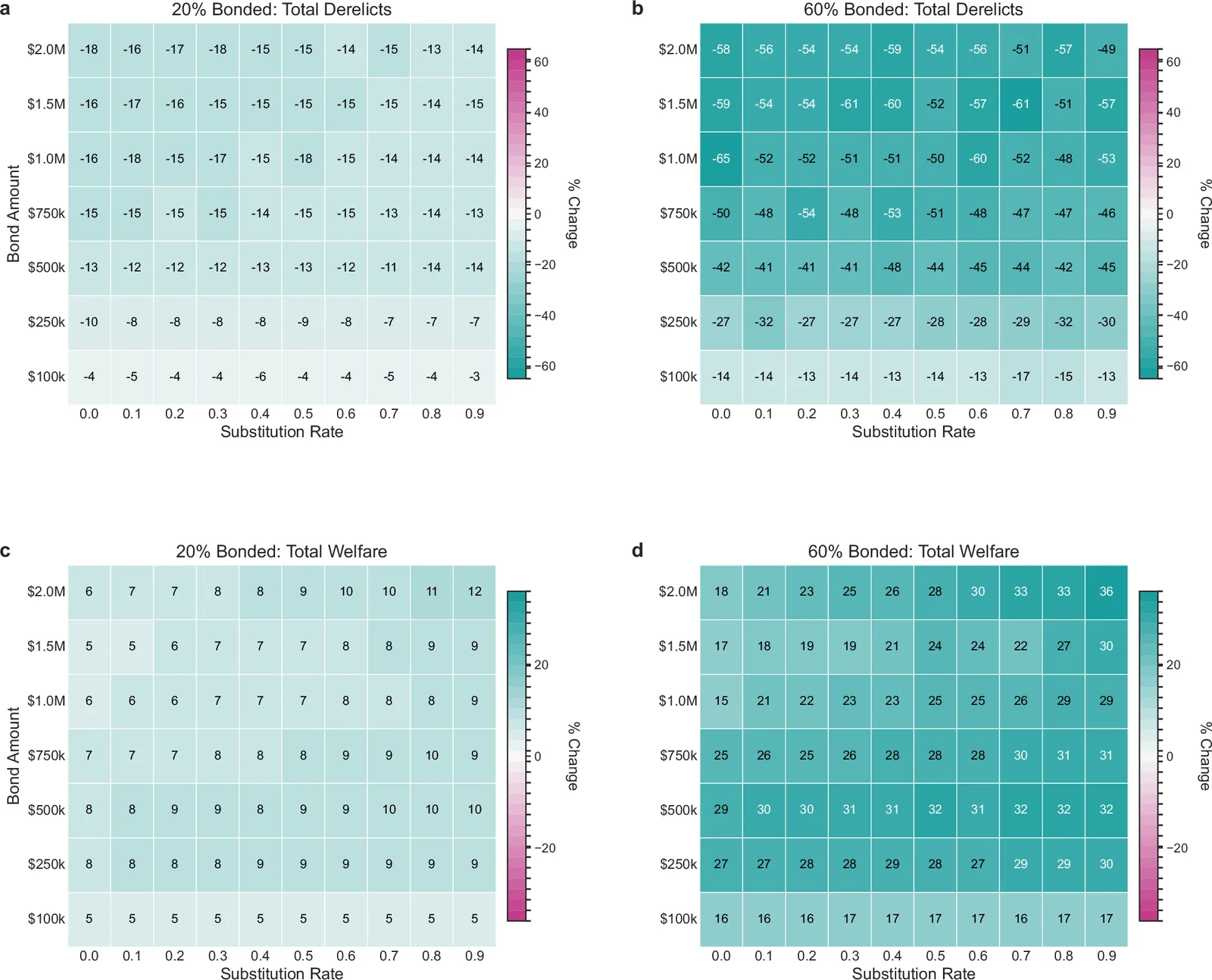
Effects of policy coverage and substitution on derelict satellites and welfare. **a**, **b** Percentage change in the combined number of derelict satellites relative to the no-policy baseline. **c**, **d** Percentage change in total welfare relative to the same baseline. The left column (**a**, **c**) presents a unilateral policy covering 20% of the market through regulation of Market A, whereas the right column (**b**, **d**) presents a multilateral policy covering 60% of the market through regulation of Markets A and B. Negative values in **a**, **b** indicate fewer derelict satellites, whereas positive values in c,d indicate greater societal economic welfare from space activities.

Individual countries or regions are likely to pursue independent policies. As an illustrative example, we explore the effects of a PMD bond policy for a satellite market that has been disaggregated into three separate markets: Market A (20% share), Market B (40% share) and Market C (40% share). Figure 4 highlights that without full bond coverage across the entire market, the bond can still reduce the number of derelicts in orbit while improving economic welfare, though outcomes are sensitive to the substitution rate across markets (see “Methods” for definition).

A bond applied only to 20% of the market (Market A—Fig. 4, left column) can achieve reductions in non-compliant derelicts of up to 18%. However, that effectiveness is eroded when there is market substitution (leakage), as the bond raises operational costs for regulated markets and market demand substitutes towards unregulated satellites with lower PMD rates. As such, expanding the regulatory coverage of the PMD bond to 60% of the market (Market A and B—Fig. 4, right column) further improves outcomes and reduces the potential for leakage, leading to reductions in derelicts on the order of 60% for larger bonds.

Total social welfare (Fig. 4, bottom row) improves in both scenarios. In the absence of market substitution, welfare increases for bonds of up to $500 k, but then declines for larger bonds as the costs of disposal and bond compliance begin to outweigh the benefits of the improved orbital environment (consistent with the single market in Fig. 2). For those larger bond amounts, where satellite operators in regulated markets are over-bonded from a welfare standpoint, substitution can actually improve welfare outcomes as satellite service demand can be met by increased launches from operators in unregulated markets. Overall, the improvements in derelict and welfare outcomes when moving from the 20% to the 60% scenario highlight that broad regulatory coordination not only protects the orbital environment more effectively, but it also enhances the long-term economic value of the sector by reducing collision risk and increasing the population of active, revenue-generating satellites.

This exercise is motivated by the fact that, in practice, it is unlikely that a PMD bond would be applied universally, because regulators in different countries must balance sustainability goals against defence and civil priorities, private-sector competitiveness, and differing incentives. Nonetheless, in a multiple market scenario, it is possible for small markets to expand a policy’s jurisdiction. As an example, the proposed EU Space Act aims to harmonise space regulation across the EU, introducing a per-satellite platform licensing fee (expected to be €100,000) and launch fees ranging from €200,000 to €1.5 million, and warns that operators should expect substantial compliance costs across environmental assessment, cybersecurity, and debris mitigation^7^. However, the EU Space Act may apply not only to EU operators but also to non-EU operators seeking access to the EU market—an extraterritorial dynamic often termed the “Brussels Effect”^21^. As with the EU’s General Data Protection Regulation and common charging standards, market access incentives can drive wider compliance beyond the regulator’s borders^22,23^.

## Discussion

PMD non-compliance in space can be framed as a governance problem of a common-pool resource system, where individual operators have economic incentives to not complete end-of-life disposal, with the resulting collision risk (and consequences) shared by all and accumulating over time. The core implication of this work is that PMD performance bonds can realign private incentives with public interest in a safe operational space environment. We indicate that bonds are effective at reducing the number of derelicts and improving space sustainability, whilst preserving and enhancing economic welfare. Furthermore, our results highlight a three-way interaction between bond size, sustainability outcomes, and welfare impacts. At smaller bond amounts, requiring direct disposal reduces collision probability and UMPY more than indirect disposal-orbit compliance, but it also imposes higher compliance costs that can reduce welfare. This is similar to findings from waste and recycling regulation that incentives should target how end-of-life management is achieved, and not just whether it occurs^24^. For bonds below $500k a sustainability-first regulator may favour direct-disposal criteria, while a welfare-first regulator may prefer the cheaper indirect disposal criteria. Finally, a PMD bond that covers only 20% of the satellite market can still drive consistent decreases in UMPY and increases in economic well-being across all market substitution rates, which is an important finding for sustainability and operational risk in LEO.

Implementing PMD bonds in practice raises design choices around verification, enforcement, and institutional capacity for policy implementation and bond financing. Verification requires both clear, unambiguous technical criteria for bond return and entities with credible capabilities to verify end-of-life outcomes. National regulations, technical standards, and industry best practices for PMD can inform technical criteria development. Similarly, a variety of governments and commercial entities possess the requisite space situational awareness and orbit propagation abilities to verify compliance against particular technical standards. Enforcement then becomes a question of political will (with third-party verification providing public accountability for both actors and regulators) as the return of the bond relies on voluntary compliance and the firm not going bankrupt during the satellite’s active lifetime^25^. We find non-compliance fees can be up to $3 billion, which could fund ADR or ISAM for orphan satellites. Implementation could be facilitated through secondary markets or existing financial infrastructure—for example, underwriting via established insurance markets such as Lloyd’s in the UK^18^. Bonds implemented through national licensing or as a condition of domestic market access are clearly compatible with national authority for authorisation and continuous supervision under the Outer Space Treaty (OST), analogous to other proposed fiscal instruments^26^.

As an alternative policy, an Orbital Use Fee (OUF) based on the collision risk in the orbital regime of operation has been considered^26^. Industry may be more inclined to support PMD bonds than an OUF, as taxes can be perceived as a net transfer from the sector, and depending on how revenues are allocated, compliant operators may feel they lose out despite meeting best practices. In contrast, bonds preserve a clearer link between behaviour and payment: operators that successfully dispose recover their contribution, while penalties fall on non-compliers, and the total revenues retained by the government could likely be smaller than under a broad-based fee. Finally, the comparative effects of an OUF versus PMD bonds on space sustainability could depend on the exact structure of the OUF. For example, an OUF imposed at launch could provide no additional incentive for proper disposal, while an OUF imposed on the entire orbital lifetime could generate incentives more similar to the PMD bond.

Other space sustainability financial mechanisms have been proposed, including launch fees^27^ and subsidies for debris remediation^17,28^. Cap and trade systems have also been considered^29^ but these may be difficult to implement, requiring techno-political consensus on an appropriate metric and threshold for measuring and trading LEO capacity, which is not universally defined^30^. While launch fees can reduce the creation of future derelicts and subsidies for debris remediation can reduce existing derelicts, PMD bonds reduce derelicts by leveraging both source reduction and increased disposal channels (analogous to deposit-refund systems for terrestrial waste^13^). More generally, debris remediation may be necessary to stabilise the long-term LEO environment, but it is a relatively expensive post-hoc measure to address existing derelicts. Importantly, the imposition of a PMD bond programme in one jurisdiction does not necessarily foreclose policy experiments in other jurisdictions or even the coexistence of multiple financial mechanisms to promote space sustainability.

Our results are subject to several important caveats in interpretation. While our model quantifies bond impacts, optimally pricing bonds remains an open challenge, subject to a variety of economic and social factors beyond the scope of our model. For instance, high compliance may not be possible if a $1–2 million bond is not sufficient to incentivize compliance from all firms or satellite types, or if some firms treat bond forfeiture as just a cost of operation^31,32^. In addition, the model necessarily abstracts from several features that could matter in practice, including heterogeneous operator risk preferences and financing costs, insurance and underwriting constraints, spectrum and licensing interactions, and stochastic failures that affect disposal capability. While we assess robustness to a variety of key factors, including bond price, refund criterion, market size, and substitution rates, coupled models inherently include many features and parameters that can be examined and refined. On the physical side, further enhancements could include modelling the launch of rocket bodies, rather than only the existing population. Furthermore, this paper evaluates space sustainability only through the orbital debris environment lens. Higher PMD compliance could increase re-entries that ablate in the upper atmosphere and may affect mesospheric temperatures and ozone^33^. Future work could assess coupled policies, such as pairing PMD bonds with incentives for low-impact re-entry materials, or a comparison to a launch tax that would internalise potential atmospheric externalities.

The findings support a clear message for policy: PMD performance bonds are a promising mechanism for improving LEO sustainability, but their design must be aligned with physical removal methods and robust to leakage under partial adoption. The broader contribution is to highlight how evaluating fiscal instruments for space requires treating LEO as a coupled system in which incentives shape behaviour and behaviour shapes environmental risk. By framing PMD as an internalisation problem with altitude-dependent physical consequences, this work helps move the policy debate beyond compliance as a binary administrative outcome and towards compliance as a measurable, environment-relevant reduction in long-lived risk. In doing so, it provides a basis for regulators to design policy instruments that can protect access to LEO for present and future generations while preserving and promoting economically valuable activity.

## Methods

All simulations are conducted within an Integrated Assessment Model (IAM) that couples a physical debris model with an economic module. In each timestep, the economic model evaluates the current debris environment and determines the optimal number of satellites to launch. The resulting launches are then propagated through the debris model, which simulates orbital evolution, collisions, and fragmentation. The two modules iterate in this way over the full simulation horizon. Each model component and its configuration are described below.

### Debris Model

**Fig. 5:**
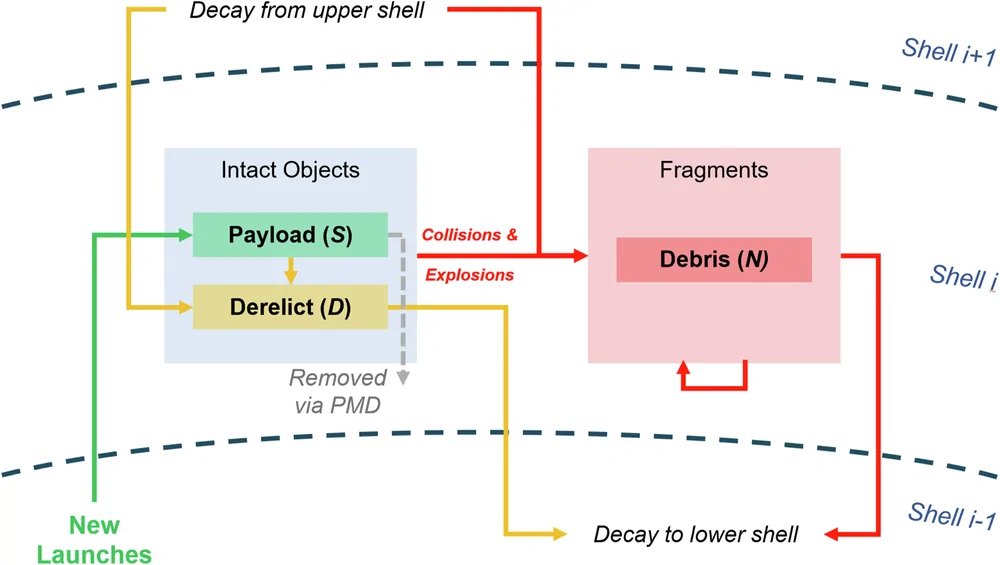
Qualitative schematic of the MIT Orbital Capacity Assessment Tool Source Sink Evolutionary Model (MOCAT-SSEM). The model divides Low Earth Orbit (LEO) into altitude shells and represents the orbital population using species corresponding to different classes of active satellites, derelict objects, rocket bodies and fragments. Population changes within each shell are determined by launches, post-mission disposal, atmospheric drag, collisions and fragmentation. In the schematic, *S*, *D* and *N* denote active payloads, derelict satellites and debris fragments, respectively. Green arrows represent new launches, the grey dashed arrow represents removal through post-mission disposal (PMD), yellow arrows represent the atmospheric decay of derelict objects, and red arrows represent collisions, explosions, fragment production and debris decay. Blue dashed curves denote the boundaries between adjacent orbital shells. Figure reproduced from^34^ under the Creative Commons Attribution 4.0 International licence.

The MIT Orbital Capacity Assessment Tool Source Sink Evolutionary Model (MOCAT-SSEM)^34^ is used as the debris model in the IAM; the model focuses on aggregate trends rather than individual object specifics. LEO is split into altitude shells and species. In this context, species refers to classes of objects, split by characteristics such as mass, radius or their object type. Examples of species could include satellites, debris or rocket bodies. Figure 5 presents the relationship between species and orbital shells, with a simplified set of species. For each orbital region, defined by an altitude range and divided into *n*_*h*_ orbital shells, each with a thickness of *d*, these objects can be classified into either sources or sinks

1 $${\dot{{{{\bf{P}}}}}}_{h}={\dot{{{{\mathbf{\Lambda }}}}}}_{h}+{\dot{{{{\bf{C}}}}}}_{h}^{{{{\rm{PMD}}}}}+{\dot{{{{\bf{F}}}}}}_{h}+{\dot{{{{\bf{C}}}}}}_{h}.$$

for a given orbital shell *h* ∈ {1, 2, ⋯ , *n*}, where the change in the population of each *P* species, $\dot{{{{\bf{P}}}}}$, is a function of launch rate, $\dot{{{{\mathbf{\Lambda }}}}}$, post-mission disposal, ${\dot{{{{\bf{C}}}}}}^{{{{\rm{PMD}}}}}$, atmospheric drag, $\dot{{{{\bf{F}}}}}$, and collisions, $\dot{{{{\bf{C}}}}}$^34^.

For each active satellite species *P*, it is typical to simulate a fraction of satellites being de-orbited from each altitude bin at each time step, based on their assumed operational lifetime, Δ*t*, to account for deliberate PMD. For the corresponding debris category associated with each active satellite type, a percentage of PMD failures is modelled to occur at each time step, reflecting the likelihood that some satellites could fail to execute their planned de-orbit procedures.

2 $${\dot{{{{\bf{C}}}}}}^{{{{\rm{PMD}}}}}=\frac{1-{P}_{{{{\rm{M}}}}}}{\Delta t}\,{{{\bf{P}}}}.$$

with a probability of success equal to *P*_M_.

Objects are binned by semi-major axis (*a*) and eccentricity (*e*), with the representative time in shell calculated as a function of their orbit to provide an effective altitude population (fractional orbits). Objects are only affected by atmospheric drag; other perturbations in LEO are ignored due to their smaller impacts on *a* and *e*. The number of *a* and *e* bins is configurable. The propagation module, eccentric collision and binning are explained in detail in ref. ^35^.

The environment model is configured to have 30 orbital shells from 200–2000 km altitude. The active satellites are split into three species: constellation satellites *S*, non-constellation satellites, *S*_u_ and CubeSats *S*_ns_. Their physical characteristics (e.g., mass) have been configured to be the same as in ref. ^34^ and are presented in SI1.

#### Disposal orbits

**Fig. 6:**
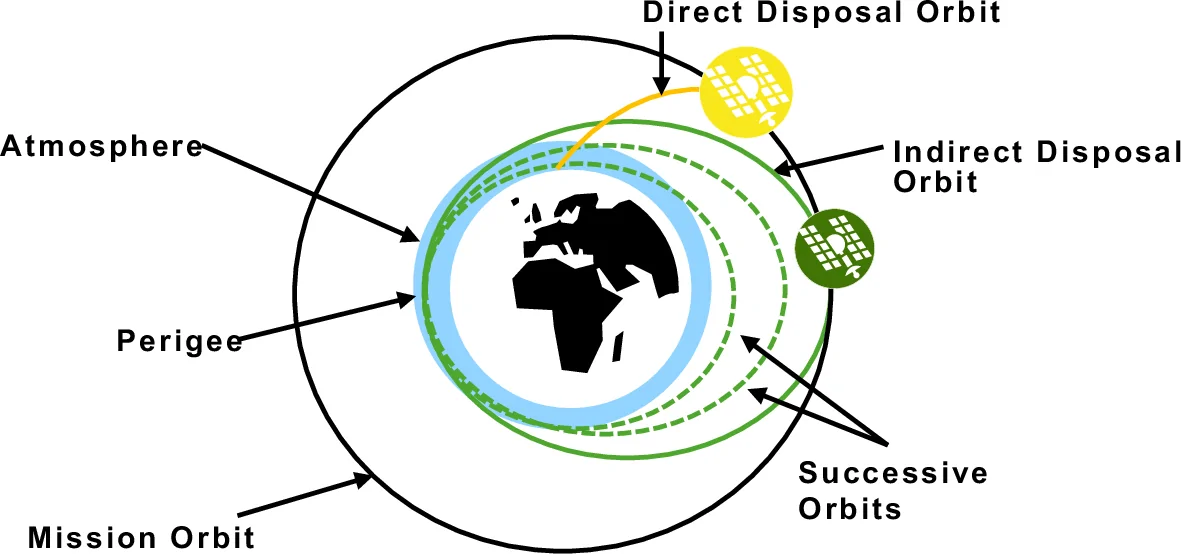
Visual description of the post-mission disposal (PMD) pathways represented in the OPUS model. The black circular line denotes the initial mission orbit, and the turquoise annulus denotes the upper atmosphere. For indirect disposal, the green satellite performs an initial manoeuvre that lowers orbital perigee; the solid green ellipse denotes the initial indirect disposal orbit, and the dashed green ellipses denote successive orbits as atmospheric drag reduces and circularises the orbit before re-entry. For direct disposal, the yellow satellite follows the yellow trajectory through the operational orbital region to controlled atmospheric re-entry over a comparatively short period. Arrows and text labels identify the mission orbit, lowered perigee, atmosphere, direct-disposal trajectory, indirect-disposal orbit and successive decaying orbits.

After the end of a satellite’s operational lifetime, four possible disposal outcomes are modelled within OPUS. A direct disposal (Fig. 6) occurs when the satellite is propulsively deorbited in less than a 1-year timestep and subsequently removed from the simulation. In a compliant indirect disposal, the satellite transitions from an active state to a derelict. If the orbit is naturally compliant within the PMD lifetime requirement, the satellite remains in its existing shell; otherwise, its perigee is lowered, and its orbit is circularised to the altitude needed to achieve re-entry within the required period (Fig. 6). The third case, no-attempt disposal, represents a scenario in which no manoeuvre is performed, and the satellite remains as a derelict in its operational orbit. Finally, a failed PMD attempt describes a situation where a disposal manoeuvre is initiated but unsuccessful, leaving the satellite as a derelict.

The perigee for a compliant indirect disposal depends on both the solar cycle and the PMD lifetime rule applied in the simulation (e.g., 5 or 25 years). The model uses the JB2008 atmospheric density model with a time-varying solar cycle derived from^36^. For each simulation year and altitude bin, the lifetime of a circular orbit is determined. If a satellite is naturally compliant, no manoeuvre is required. For non-compliant satellites, following the method of^37^, it is assumed that operators perform the minimum manoeuvre necessary to meet the PMD requirement. A single burn is executed at apogee to reduce the perigee to an altitude that guarantees re-entry within the specified lifetime. The satellite then transitions to this new orbit, where it gradually circularises and decays, with re-entry defined as a perigee altitude below 150 km. For a direct disposal, more propellant is required to dispose straight into orbit, reducing the operational lifetime of the satellite.

Disposal orbits for each initial condition can be precomputed using MOCAT Monte Carlo (MC)^38^, a higher fidelity orbital debris model that propagates objects individually. For each satellite type (*S*, *S*_u_, and *S*_ns_), assuming an initially circular orbit, apogee and perigee altitudes are sampled every 50 km across low Earth orbit. Using MOCAT-MC with a five-day timestep, the perigee altitude required to achieve a target lifetime is calculated for each year from 2024 to 2050. The resulting dataset is used to generate interpolated functions for each satellite type and year. These functions are then exported to OPUS, allowing the model, at runtime, to input the satellite type, starting year, and desired lifetime and return the corresponding perigee altitude required for disposal. As a visual example, see SI Fig. S7, which highlights how the orbital decay lifetime for *S*_u_ and *S*_ns_ on a circular orbit with a semi-major axis of 7371 km. Results are presented for two start years—near solar maximum in 2025 and near solar minimum in 2034.

### Economic model

The economic model within OPUS considers the launch decisions of a representative operator who accounts for the orbital environment when choosing whether to launch a satellite of species *i* to orbital altitude *h* at time *t*. In similar coupled models^20,39,40^, only one type of satellite was considered, and constellation satellites were exogenously launched independent of any endogenous economic incentives^39^. The enhanced model now supports the simultaneous launch of multiple satellite species, each with distinct characteristics. Each species launches without knowledge of other satellites deployed within the same timestep, but takes into account all objects present from previous timesteps. Below, the notation is for a single species of satellite, while the model is solved at each timestep, for each satellite species.

Forward-looking operators, when considering a launch, must account for market demands and potential revenues, as well as lift and disposal costs (increasing in *h*) and station-keeping and drag (decreasing in *h*). Firms must also account for the risk of a collision — let ${P}_{h}\left({{{{\bf{S}}}}}_{t}^{{{{\rm{A}}}}},{{{{\bf{D}}}}}_{t}\right)$ be the probability that a firm assigns to the risk of collision, which depends on the vectors of active satellite ${S}_{t}^{A}$ and debris *D*_*t*_ counts—a key input from MOCAT-SSEM described above. Satellites are assumed to have a lifetime of 1/*δ*, where *δ* is the depreciation rate of the satellite. Given the open access environment operators could add ${\hat{X}}_{ht-1}$ satellites (new launches) of a given species to location *h* until zero equilibrium profits are achieved in period *t*:

3 $$\forall h,\,{\hat{X}}_{ht-1}:{R}_{h}({S}_{t})-r-\delta -(1-\delta ){P}_{h}({{{{\bf{S}}}}}_{t}^{{{{\rm{A}}}}},{{{{\bf{D}}}}}_{t})=0.$$

The gross rate of return on a satellite is given by *R*_*h*_(*S*_*t*_) = *p*(*S*_*t*_)/*c*_*h*_, where *p*(*S*_*t*_) is the per-period revenue earned by a satellite of a specific species type, and *c*_*h*_ is the cost to launch and operate a satellite at altitude *h*. ${R}_{h}({{{{\bf{S}}}}}_{t}^{{{{\rm{A}}}}})$ is assumed to be decreasing in total satellites of a given species *S*_*t*_, $\frac{d{R}_{h}}{d{S}_{t}} < 0$, due to downward sloping demand for satellite services. The number of satellites launched to an altitude depends on the gross rate of return on satellite services at that location *R*_*h*_(*S*_*t*_), the opportunity cost of funds (the interest rate) *r*, depreciation of the satellite *δ*, and the probability of collision ${P}_{h}({{{{\bf{S}}}}}_{t}^{{{{\rm{A}}}}},{{{{\bf{D}}}}}_{t})$. See SI Table S1 for a breakdown of the costs for each species.

When Eq. (3) holds, the marginal operator is exactly indifferent between launching and not launching an additional satellite. Equation (3) thus reflects the open access equilibrium where profitable altitudes are filled by launches, updating the number of satellites at a given altitude *S*_*ht*_ until the combination of downward-sloping demand and increasing collision risks drives profits to zero.

The gross rate of return has two components: the expected future revenues that the satellite generates in period *t*, *p*(*S*_*t*_), and the annualised unit cost of deploying it to an orbital shell, *c*_*h*_. The downward sloping demand curve *p*(*S*_*t*_), representing the willingness-to-pay for satellite services, depends on the species. The demand curve is calibrated by using simulated launches from 2016 to determine the revenue required to match the observed number of satellites of each species in 2025 (see ^35^ and SI2 for additional details).

The annualised unit cost *c*_*h*_ reflects three main factors^39^. Firstly, the lift price *c*_lift_ is the dollar cost per kilogram of accessing LEO, multiplied by the mass of the satellite payload of each species. Secondly, the cost of the manoeuvreing budget (delta-v) for a given altitude *c*_Δ*v*_(*h*). This is the cost of delta-v required to 1) maintain a satellite in its target orbit and 2) conduct any necessary manoeuvres over its lifetime, which can depend on the probability of collision at that altitude *h*. The final factor is the opportunity cost of foregone operational lifetime from deorbiting a satellite from altitude *h* to altitude h^*^, *c*_*μ*_(*h*, h^*^). This is the opportunity cost of a satellite’s lifetime being reduced by expending propellant to deorbit, and is the key cost term for PMD bonds. Letting the rate of compliance with deorbit regulations be *C*_*h*_, the complete cost function, for an altitude of *h* given a target deorbit location h^*^ is:

4 $${c}_{h}={c}_{{{{\rm{lift}}}}}+{c}_{\Delta v}(h)+{C}_{h}{c}_{\mu }(h,{h}^{*}).$$

#### Bond implementation

We operationalise Adilov et al.’s^19^ first proposition by deriving the minimum value of the bond, ${b}_{h}^{*}$, needed to fully incentivise a satellite in orbital shell *h* to deorbit after their active lifetime, Δ:

5 $${b}_{h}^{*}=A\left[\frac{1-{\beta }^{{{{{\mathbf{\Lambda }}}}}_{h}}{(1-w)}^{{{{{\mathbf{\Lambda }}}}}_{h}}}{1-\beta (1-w)}\right]{\beta }^{\Delta }(1-w).$$

6 $$=A\left[\frac{1-{\beta }^{{{{{\mathbf{\Lambda }}}}}_{h}}}{1-\beta }\right]{\beta }^{\Delta }.$$

where the discount factor *β* accounts for opportunity cost of time and is related to the discount rate via $\beta=\frac{1}{1+r}$. To account for foregone revenues due to deorbiting, *A* is the maximum per-period willingness-to-pay for services from a satellite, defined and calibrated (see SI3). **Λ**_*h*_ is the opportunity cost of deorbiting from *h*, expressed in years of foregone mission lifetime due to deorbiting. Δ is the mission lifetime (expressed in years and equal to 1/*δ*). Adilov et al. allow for a non-zero failure rate of a satellite; *w*, however, here we assume no random failures during active lifetime (*w* = 0). Therefore, the value of ${b}_{h}^{*}$ can be simplified to Eq. (6), representing the bond amount such that a satellite operator profits more from proper disposal and bond-recovery than from leaving the satellite in orbit.

Whereas Adilov et al.’s^19^ bond derivation features a binary comply/do not comply structure, in reality we observe varying rates of deorbiting compliance, even without bonds in place. For reasons discussed in further detail in SI5, the relationship between the rate of PMD compliance for non-naturally compliant orbits, *C*_*h*_(*b*), and the dollar cost of the bond can be expressed as:

7 $${C}_{h}(b)=\left\{\begin{array}{ll}0.97-\xi \cdot \exp \left(-k\cdot \frac{b}{{b}_{h}^{*}}\cdot 100\right),\quad & \,{\mbox{if}}\,\,b\ne 0\\ {P}_{{{{\rm{M}}}}},\quad & \,{\mbox{if}}\,\,b=0\end{array}\right.$$

where *ξ* = 0.52, $k=\frac{\,{\mbox{ln}}\,(12)}{75}$, ${b}_{h}^{*}$ is the critical bond amount defined above, and *P*_M_ is the historical baseline of PMD in the absence of bonds. Note that lim_*b*→0_*C*_*h*_(*b*) = *P*_M_ and see SI Fig. S8 for the asymptotic relationship between the cost of the bond and compliance to the policy.

Bonds affect equilibrium outcomes directly through launch incentives and indirectly through changes in the space environment. PMD compliance raises deorbiting costs through Eq. (4) as well as reduces the return on launch for non-compliers due to foregone bond revenues ((1 − *C*_*h*_(*b*))*b*). When bond amount *b* is applied, the gross rate-of-return can be expressed as *R*_*h*_(*S*_*t*_) = *p*(*S*_*t*_)/(*c*_*h*_ + (1 − *C*_*h*_(*b*))*b*). While the bond reduces the gross rate of return on launching relative to the no-policy equilibrium described in Eq. (3), the bond also incentivises deorbiting compliance and the reduction of derelicts, reducing the probability of collision ${P}_{h}({{{{\bf{S}}}}}_{t}^{{{{\rm{A}}}}},{{{{\bf{D}}}}}_{t})$ and encouraging more launches into the cleaner space environment.

#### Market substitution

To examine market substitution and leakage, the satellite market is disaggregated into bonded and unbonded categories, a classification that determines both the internal cost structure of the operator and the specific disposal logic applied to the satellite.

To illustrate the effects of regulatory leakage, the model incorporates a reduced-form and intuitive substitution mechanism wherein reductions in launches in the regulated market due to the bond’s fiscal burden are fractionally replaced by increases in launches in unregulated markets. To operationalise this mechanism, the substitution rate in Fig. 4 simply captures the share of new launches in unregulated markets per reduction in launches in the regulated region. For example, if the substitution rate is 0.5, then if the bond reduces launches by 50 satellites in the regulated market, demand parameters in the unregulated market are adjusted to produce 25 new satellite launches. This is done separately for each species to avoid unlikely substitution pathways between distinct satellite service markets.

The simulation considers three distinct markets (or regions)—A, B, and C—representing market shares of 20%, 40%, and 40%, respectively. All simulations cover a 25-year horizon under a 5-year PMD rule, and consider market substitution rates from 0 to 0.9 (in 0.1 increments). Two policy configurations are analysed: a unilateral policy where the bond is applied solely to Market A (20% of the global market), and a multilateral policy where the bond is applied to Markets A and B (60% of the global market). In either case, bonded operators are subject to the compliance incentives described in Eq. (7).

We simulate the full range of substitution rates, as the substitution rate across different regional or national satellite markets is difficult to determine and has not been published for space-based services. As Hillberry et al.^41^ note, a key question in trade research is how demand for domestic versus foreign goods responds to changes in relative prices, and note that the resulting elasticity is the most important parameter in modern trade theory. Furthermore, the size of the elasticity used for calibration is often critical for model conclusions^42^. In general, across a wide variety of goods, empirical trade studies find that domestic and foreign goods are imperfect substitutes for each other, suggesting that the substitution rate may be closer to 0 than 1 for the space sector. Furthermore, space assets are closely aligned with national strategic interests, tightly regulated through licensing and spectrum coordination, and often embedded in long-term procurement and service contracts that are not easily reallocated across jurisdictions in response to modest changes in relative costs, indicative of lower substitution rates.

All code is included in the OPUS Github and Zenodo repository, along with readme files detailing specific model runs.

### Metrics for sustainability and welfare

To evaluate a policy’s efficacy for the long-term sustainability of LEO, we use a proxy related to the total mass of debris that is added to the environment and its orbital lifetime: Undisposed Mass Per Year (UMPY). It assesses the total amount of mass, as a function of orbital lifetime^43^. UMPY is calculated as:

8 $${{{\rm{UMPY}}}}=\frac{1}{{t}_{{{{\rm{sim}}}}}}{\sum}_{i=1}^{{n}_{{{{\rm{objs}}}}}}{m}_{i}\left[\frac{\exp \left[X\left(\frac{{{{{\rm{life}}}}}_{i}}{{t}_{{{{\rm{sim}}}}}}-1\right)\right]-1}{\exp (X)-1}\right].$$

where ${t}_{{{{\rm{sim}}}}}$ (in years) denotes the total duration of the study or simulation and *n*_objs_ represents the number of uncontrolled objects in LEO. Each object *i* has a mass *m*_*i*_ (in kilograms), and a lifetime life_*i*_ (in years), which is the period it remains uncontrolled before reentry or removal, which is provided from MOCAT-SSEM. Finally, *X* is a dimensionless lifetime scaling exponent that modifies the effect of life_*i*_ within the exponential term. We use *X* = 4 as suggested by ref.^43^.

In addition, total economic welfare is a metric that captures the total economic benefit from space activities, and thus can be used to determine the consequences (positive or negative) for the space economy from policies pursuing environmental goals. It consists of both consumer surplus and producer surplus, plus any net government revenues. Because of open access (Eq. (3)), satellite operators launch until producer surplus is fully dissipated^20^. This leaves consumer surplus - the difference between what consumers are willing to pay for satellite services and the actual price they pay - and bond revenue as the determinants of welfare.

Given a linear demand function of the form, *p*(*S*_*kt*_) = *A*_*k*_ − *B*_*k*_*S*_*kt*_ for species *k* at time *t*, economic welfare for a given bond policy *b* is then:

$${{{\mathcal{K}}}}=\left\{S,\,{S}_{{{{\rm{u}}}}},\,{S}_{{{{\rm{ns}}}}}\right\}.$$

9 $${{{\rm{Welfare}}}}(t)={\sum}_{k\in {{{\mathcal{K}}}}}\frac{1}{2}{B}_{k}{S}_{k,t}{(b)}^{2}+{\sum}_{k\in {{{\mathcal{K}}}}}{\sum}_{h\ge {h}^{{{{\rm{NC}}}}}}\left[1-{C}_{h}(b)\right]b\,{S}_{k,h,t}(b).$$

representing the standard triangle of consumer surplus plus any net revenue retained by the government from non-compliant satellites in non-naturally compliant orbits (those above *h*^NC^).

### Reporting summary

Further information on research design is available in the Nature Portfolio Reporting Summary linked to this article.

## Supplementary information

Supplementary Information

Reporting Summary

Transparent Peer Review file

## Data Availability

All data generated and analysed in this study have been deposited in the Zenodo repository and are openly available at https://doi.org/10.5281/zenodo.20506352.

## Code availability

The custom code used to generate and analyse the results presented in this study has been deposited in Zenodo and is publicly available at https://doi.org/10.5281/zenodo.20506352. The archived repository contains the scripts required to reproduce the analyses and figures reported in the manuscript. No restrictions apply to access.
