# Supplementary material for: Bonds improve post mission disposal compliance and the sustainability of low Earth orbit
Source: Nat Commun. 2026 Sep 15;17:10836. doi: 10.1038/s41467-026-77660-4 (PMC13672303; doi:10.1038/s41467-026-77660-4)
Supplement: Supplementary file 1 [file 41467_2026_77660_MOESM1_ESM.pdf]

Supplementary Information

Bonds Improve Post Mission Disposal Compliance and the Sustainability of Low Earth Orbit

I. Brownhall[1,*], D. Kaffine[2], D. Kilpatrick[2], M. Lifson[3], M. Moretto[4], E. Stevenson[5], S. Lemmens[6,], F. Letizia[6], and S. Bhattarai[1]

[1]University College London (UCL), OrbitLab, Department of Engineering, UK. Emails: indy.brownhall@gmail.com; s.bhattarai@ucl.ac.uk.

[2]University of Colorado Boulder, Department of Economics, Renewable and Sustainable Energy Institute, USA. Emails: daniel.kaffine@colorado.edu; joey.kilpatrick@colorado.edu.

[3]The Aerospace Corporation, USA. Email: miles.t.lifson@aero.org.

[4]North Carolina State University, Department of Mechanical and Aerospace Engineering, USA. Email: mjmorett@ncsu.edu.

[5]IMS Space Consultancy at ESA ESTEC, The Netherlands. Email: emma.stevenson@ext.esa.int.

[6]European Space Agency, ESTEC, The Netherlands. Emails: stijn.lemmens@esa.int; francesca.letizia@esa.int.

[*]Corresponding author: indy.brownhall@gmail.com.
[†]Joint supervision.

Supplementary items

Supplementary Methods 1. MOCAT-SSEM description 3
Supplementary Methods 2. Compliance 5
Supplementary Methods 3. Revenue estimation 5
Supplementary Note 1. Species-level effects of the bond 6
Supplementary Note 2. PMD compliance curve 6
Supplementary Figure 1. MOCAT-SSEM model schematic 8
Supplementary Figure 2. Flux-model methodology 9
Supplementary Figure 3. Observed non-naturally compliant satellites 9
Supplementary Figure 4. Active-satellite populations in 2017 and 2025 10
Supplementary Figure 5. Species-level outcomes under PMD bonds ??
Supplementary Figure 6. Orbital lifetime and disposal-orbit perigee 10
Supplementary Figure 7. PMD compliance curve 11
Supplementary Figure 8. Economic welfare under direct and indirect disposal 11
Supplementary Table 1. MOCAT-SSEM and OPUS parameters 12
Supplementary Table 2. Observed PMD outcomes by species 12
Supplementary Table 3. Calibrated revenue parameters 12
Supplementary Table 4. Cumulative non-compliance revenue 13
Supplementary References. 14

Figure-reporting scope. Supplementary Figures 1 and 2 are conceptual schematics. Supplementary Figures 4 and 6–9 present deterministic model outputs, for which biological sample sizes, error bars, inferential statistical tests and p-values are not applicable. Supplementary Figures 3 and 5 present complete population datasets, with exact counts stated in their legends. No gels, blots, micrographs, molecular-weight markers or representative experimental images are included; repeat counts and uncropped scans are therefore not applicable.

Supplementary Methods 1: MOCAT-SSEM description

The model focuses on aggregate trends rather than individual object specifics. LEO is split into altitude *shells* and *species*. In this context, species refers to classes of objects, split by characteristics such as mass, radius or their object type. Examples of species could include satellites, debris or rocket bodies. Figure 1 presents the relationship between species and orbital shells, with a simplified set of species. For each orbital region, defined by an altitude range and divided into n_h orbital shells, each with a thickness of d, these objects can be classified into either *sources* or *sinks*

$$\dot{\mathbf{P}}_h = \dot{\mathbf{\Lambda}}_h + \dot{\mathbf{C}}_h^{\mathrm{PMD}} + \dot{\mathbf{F}}_h + \dot{\mathbf{C}}_h \tag{S1}$$

for $h_i \in \{1, 2, \cdots, n_h\}$, where the change in the population of each P species, $\dot{\mathbf{P}}$, is a function of launch rate, $\dot{\mathbf{\Lambda}}$, post-mission disposal, $\dot{\mathbf{C}}^{\mathrm{PMD}}$, atmospheric drag, $\dot{\mathbf{F}}$, and collisions, $\dot{\mathbf{C}}$ [1].

For each active satellite species P, it is typical to simulate a fraction of satellites being de-orbited from each altitude bin at each time step, based on their assumed operational lifetime, Δt, to account for deliberate post-mission disposal. For the corresponding debris category associated with each active satellite type, a percentage of post-mission disposal failures is modelled to occur at each time step, reflecting the likelihood that some satellites will fail to execute their planned de-orbit procedures.

$$\dot{\mathbf{C}}^{\mathrm{PMD}} = \frac{1 - P_{\mathrm{M}}}{\Delta t}\,\mathbf{P} \tag{S2}$$

with a probability of success equal to P_{M}.

Objects are binned by semi-major axis (a) and eccentricity (e), with the representative time in shell calculated as a function of their orbit to provide an effective altitude population (fractional orbits). Objects are only affected by atmospheric drag, other perturbations in LEO are ignored due to their smaller impacts on accelerations and position. The number of a and e bins is configurable.

Collisions

A fundamental element of any debris model is calculating the probability of collision and the consequent number of fragments. In the SSEM framework, each species is uniquely paired with others, representing the set of potential collisions pairs in the simulation. The collision rate for species i denoted as $\dot{C}_i$ is given by:

$$\dot{C}_i = \sum_{k=1}^{N_{\mathrm{s}}} \Gamma_{ij}\phi_{ij}P_iP_j + \dot{C}_{i,\mathrm{add}} \tag{S3}$$

This equation indicates that in each shell and for each collision species pair, the primary and secondary collision species lose objects to collisions at a rate based on the behaviours of the two species Γ_{ij} and the physical geometry of the objects and shells ϕ_{ij}. For debris species, the population is potentially also increased by the output of collisions between other species pairs, $\dot{C}_{i,\mathrm{add}}$.

Γ_{ij} is a matrix that quantifies the likelihood of a collision between species P_i and P_j, generally beginning with a value of -1, to reflect that the species in that shell is decremented by one during a collision, and additional terms that modify collision probability to reflect factors like orbit coordination for mutual physical separation and collision avoidance manoeuvrer efficacy. For example, in the case of two manoeuvrable slotted satellites species Γ_{ij} may be set to zero under the assumption that two satellites within the same constellation will not collide.

$$\phi_{ij} = \pi \frac{v_{\mathrm{r}}(h)\sigma_{ij}}{V(h)} \tag{S4}$$

Equation S4 represents the physical aspects of collision probability subject to a kinetic theory of gases assumption. $V(h)$ and $v_{\mathrm{r}}(h)$ are the volume of the altitude shell and the average velocity of the shell. σ_{ij} is a function of radius of the colliding objects, r_i and r_j:

$$\sigma_{ij} = (r_i + r_j)^2 \tag{S5}$$

Finally, $\dot{C}_{i,\mathrm{add}}$ represents the additional fragments generated from a collision (n_{f}) based on its impact velocity. For each collision, if the impact velocity is above $40\,\mathrm{J\,g^{-1}}$ then it is considered to be catastrophic. The number of fragments are calculated from the NASA Standard Break Up Model [2] and then are binned back to each species by their mass.

The number of manoeuvrers are challenging to estimate in a SSEM due to the lack of an orbit trajectory and the theory of gas assumption. Therefore, the model applies a baseline assumption that manoeuvrable satellites experiences the same number of potential collision events as other species before scaled by $\Gamma_{i,j}$. This corresponds to an approximate one-to-one ratio between true collisions and avoidance manoeuvres. The frequency of such manoeuvres can also be

scaled: for example, applying a factor of two implies that, on average, a manoeuvrable satellite performs two avoidance manoeuvres for every one true collision.

Species

The debris model is configurable to any number of species and orbital shells. For this paper, we following the classification proposed by [3], we include three satellite types: constellation satellites (S), non-constellation satellites (S_{u}), and satellites below 20 kg (including CubeSats) (S_{ns}). The debris population has been split into debris and rocket bodies. Only debris greater than 10 cm is modelled, there are 3 active, 6 debris species and 1 rocket body species. Table 1 summarises the complete configuration of all species used in the MOCAT–SSEM debris environment model and the OPUS economic module. These parameters define the physical characteristics (mass, radius, drag coefficient), operational behaviour (manoeuvrability, PMD success rates, slotted status), and the economic attributes used in the IAM (revenue intercepts, delta-v costs, disposal preferences).

Elliptical Orbits

MOCAT-SSEM uses a flux model that uses the Brouwer-Lyddane theory [4, 5] to average perturbations to obtain the variational equations for the orbital elements with the combined effect of drag and oblateness. The propagator assumes that the density is constant at each time step and the orbit eccentricity is small. To reduce the computational effort, only the effect of atmospheric drag is modelled to derive the rate of change for the semi-major axis a and eccentricity e. These are then used in the updated SSEM equations to provide the time-dependent transition of objects from particular a/e bin to other a/e bins.

Let us define:

$$\beta_0 = \frac{\sqrt{3}}{2}\,\bar{e}_0, \qquad \bar{n}_0 = \sqrt{\frac{\mu}{\bar{a}_0^3}}, \qquad C_0 = \frac{1}{2} C_{\mathrm{D}} \frac{A}{m} \rho_0; \tag{S6}$$

the resulting set of equations used for the propagation is

$$\begin{aligned} \frac{\mathrm{d}\bar{a}}{\mathrm{d}t} &= -\left(\frac{2\bar{a}_0^2 \bar{n}_0 C_0}{\beta_0 \bar{e}_0}\right) \tan\left(\operatorname{atan}(\beta_0) - \beta_0 \bar{n}_0 \bar{a}_0 C_0 (t - t_0)\right) \sec^2\left(\operatorname{atan}(\beta_0) - \beta_0 \bar{n}_0 \bar{a}_0 C_0 (t - t_0)\right) \\ \frac{\mathrm{d}\bar{e}}{\mathrm{d}t} &= -\bar{e}_0 \bar{n}_0 \bar{a}_0 C_0 \left(\frac{1}{\cos\left(\operatorname{atan}(\beta_0) - \beta_0 \bar{n}_0 \bar{a}_0 C_0 (t - t_0)\right)}\right)^2 \end{aligned} \tag{S7}$$

where $\bar{a}, \bar{e}$ represents the averaged semi-major axis and eccentricity of the space object at time t. The subscript 0 denotes the state variables at the initial time of propagation t_0, μ the gravitational parameter, ρ the atmospheric density, and C_{D}, A, and m representing the drag coefficient, area, and mass, respectively. The atmospheric density is calculated from JB2008 [6] with a solar cycle added [5], LEO atmospheric density over time is shown in Figure **??**.

The second key change is the movement of objects across bins by assessing the rate of change of objects into bins through a flux. First, the model defines a set number of bins for a and e. Figure 2 presents a visual description of the method. Grid cells or boxes are defined in both a and e. After the grid cells are derived, the rate of change of the objects flowing into each cell is required, $\dot{a}$ and $\dot{e}$. The next step to is to use those to find the flux across each boundary. As the box walls are orthogonal in a and e, the flux will functionally going in a and e directions. The model forces that these orbital elements will only decrease.

The density of objects in each cell $N(a, s, e)$ is initialised from a given population distribution. Each grid cell of size $\Delta a \times \Delta e$ in semi-major axis and eccentricity space, is indexed by the pair (a, e), where a and e denote the bin-centred values of the semi-major axis and eccentricity, respectively. The total number of objects in each cell is given by $N(a, s, e)$ for species P at time t. The time evolution of $N(a, s, e)$ is governed by the net flux of objects across cell boundaries, which is computed from the analytical orbit propagator that provides the change of $\dot{a}(a, e)$ and $\dot{e}(a, e)$ due to atmospheric drag. Using a finite-volume approach, the change of object in a cell (a, e) is given by:

$$F(a, P, e) = \Phi^a_{\mathrm{in}}(a, P, e) - \Phi^a_{\mathrm{out}}(a, P, e) + \Phi^e_{\mathrm{in}}(a, P, e) - \Phi^e_{\mathrm{out}}(a, P, e) \tag{S8}$$

where Φ^a_{in} and Φ^e_{in} represent the flux entering from adjacent higher a and e bins, while Φ^a_{out} and Φ^e_{out} are the fluxes leaving the current cell. The outgoing fluxes are computed as:

$$\Phi^a_{\mathrm{out}}(a, P, e) = \frac{N(a, P, e) \cdot \dot{a}(a, e)}{\Delta a} \tag{S9}$$

$$\Phi^e_{\mathrm{out}}(a, P, e) = \frac{N(a, P, e) \cdot \dot{e}(a, e)}{\Delta e} \tag{S10}$$

These represent the number of objects leaving the cell per unit time due to change in a and e, normalized by the size of the bin in each dimension. Since the total population in a cell cannot become negative. This method has been validated in [7].

Supplementary Methods 2: Compliance

For each satellite species, the baseline level of PMD compliance is estimated using historical compliance data from 2000–2023, as reported in the ESA Space Debris Report [8]. Since the dataset does not distinguish between satellite types, the counts for each category have been inferred based on whether a satellite forms part of a constellation and its mass. OPUS models all satellites that are naturally compliant as assumed to achieve PMD success with no failure. Figure 3 illustrates the annual compliance levels, divided into the four disposal outcome categories described previously.

Table 2 summarises the average compliance levels for each satellite species, calculated over two time periods: 2000–2023 and from 2010 onwards. The results indicate that compliance among non-naturally compliant satellites has improved in recent years; however, the proportion of satellites with no deorbit attempt remains high—58.5% for S and 37.7% for S_{u}. Providing a baseline average of **45%** compliance for satellites with manouevering capability in non-naturally compliant orbits. If S_{ns} are included, this reduces to 40%.

Interestingly, S_{u} exhibits a higher proportion of successful direct disposals compared to S. As S_{ns} satellites are non-manoeuvrable, they consistently show 100% in the no-deorbit-attempt category.

The OPUS model projects the evolution of PMD compliance over the next 25 years. Constellations continue to be deployed, improvements in propulsion technology and reductions in manoeuvre costs are expected to enable greater compliance among satellites operating in non-naturally compliant orbits, particularly for S-type satellites. Consequently, S satellites are assumed to achieve higher active disposal rates, with 90% completing a controlled direct disposal and the remaining 10% failing to deorbit. This reflects the expectation that uncontrolled disposals will become increasingly rare, as they carry the risk of a derelict satellite briefly re-entering the operational altitude band during early orbital decay. For example, OneWeb, which operates at approximately 1,200 km altitude, had launched 654 satellites as of October 2025, with only two failures recorded. As an increasing proportion of LEO traffic consists of satellites from large, professionally managed constellations, compliance is expected to improve further due to a combination of self-interested operational incentives and growing normative, political, and technical pressures to ensure responsible end-of-life behaviour.

For S_{u} satellites, representing smaller or individually operated spacecraft, compliance improvements are expected to be more limited. These satellites typically have fewer operational constraints and less coordination with other systems, which reduces the incentive for active disposal. Therefore, the proportion of successful direct disposals for a no bond scenario is assumed to remain at 65%, with around 35% undergoing uncontrolled re-entry.

Finally, for S_{ns} satellites, it is anticipated that manoeuvring capabilities will become increasingly common, even among small and CubeSat satellites, due to continued miniaturisation and cost reductions in propulsion technology. The proposed EU Commission Space Act has also argued for mandatory propulsion on all satellites that are operated in, or sell products to the European Union. In a policy context where an orbital bond or similar economic incentive is introduced, we assume that non-manoeuvrable satellites are less likely to be deployed. As such, S_{ns} satellites are assigned the same baseline PMD configuration as S_{u}, but with a reduced bond value to reflect their lower mass and economic impact.

Supplementary Methods 3: Revenue estimation

The amount of revenue a satellite generates for one year in orbit is extremely difficult to estimate. In principle, revenue could be estimated from public market-based data, but companies often only report total revenue rather than breaking it down per satellite. Moreover, many companies provide value-added products incorporating detail from multiple sources, again complicating the ability to connect revenue to particular satellites. These parameters can be estimated through other methods, including first-principles engineering models (e.g., [9]) and empirical inference from observed historical launch behaviour [10].

Lacking ideal economic data, we have used the approach outlined by [7] by calibrating OPUS economic parameters against historical launch data. For simplicity, the inverse demand function representing willing-to-pay for satellite services from species i is assumed to be linear:

$$p_i(S_{i,t}) = a_i + b_i S_{i,t} \tag{S11}$$

We treat the baseline demand curve intercepts a_i (one per satellite type i) and slope coefficients (b_i) as unknowns and optimise over them to match observed counts from 2017 to 2025. Intuitively, these represent (i) the maximum customers would pay annually for satellite services absent any competition, and (ii) the loss in revenue per satellite resulting from competition as other satellites accumulate.

Revenue is set exogenously by the optimiser and passed into the model; the elasticity is then set based on that revenue value and is also supplied exogenously. The elasticity is the percentage change in satellite service demand for species i for a 1% change in price, p_i:

$$\epsilon_{S_i,p_i} \equiv \frac{\mathrm{d}S_i}{\mathrm{d}p_i}\frac{p_i}{S_i} < 0, \tag{S12}$$

where S_i is the total number of satellites. Intuitively, the elasticity captures the response of demand to changes in prices - if the price of satellite services increased by 30% overnight, how much demand for satellite services would there be? If $|\epsilon_{S_i,p_i}| < 1$ demand is relatively inelastic (small change in S_i for a given change in p_i); if $|\epsilon_{S_i,p_i}| > 1$ it is relatively elastic (large change in S_i. Assuming a linear inverse demand curve (Equation S11) the elasticity becomes

$$\epsilon_{S_i,p_i} = -\frac{a_i - b_i S_i}{b_i S_i}. \tag{S13}$$

To our knowledge, no empirical elasticity estimates exist in the wider literature for satellite communications. As such, we follow [11], which finds the (terrestrial) telecommunications demand elasticity to be roughly -1, equating to a 1% increase in price will lead to a 1% decrease in quantity demanded. Imposing $\epsilon_{S_i,p_i} = -1$ in (S13) gives:

$$-1 = -\frac{a_i - b_i S_i}{b_i S_i} \quad \Rightarrow \quad b_i = \frac{a_i}{2S_i}. \tag{S14}$$

Let $\mathcal{I} = \{S, S_{\mathrm{u}}, S_{\mathrm{ns}}\}$ be the set of satellite species with endogenous demand. Starting OPUS on 1st January 2017, we choose $\mathbf{a} = \{a_i\}_{i\in\mathcal{I}}$ to match the observed (target) number of satellites N_i^{target}:

$$\min_{\mathbf{a}} \sum_{i\in\mathcal{I}} \left(N_i^{\mathrm{OPUS}}(\mathbf{a}) - N_i^{\mathrm{target}}\right)^2, \tag{S15}$$

The Union of Concerned Scientists (UCS) Satellite Database [12] was used to determine the number of each LEO satellite species launched before 1st January, 2017. The database was first filtered for only "Commercial" users, to remove military and governmental satellites[1], as these will follow different revenue models and OPUS is not designed to accommodate these types of satellite. CubeSats were differentiated by a dry mass less than 20kg. Constellations were determined using constellation names, including GlobalStar, Iridium and OrbComm. In 2025, the same process was applied. Space-Track.org information was used to augment the UCS database, which was last updated in 2023. In 2017 there were 21 S, 22 S_{u} and 2 S_{ns} (see).

OPUS was configured with the correct starting initial satellite population and their altitude. No non-tracked debris was included in the initial population. Altitudes of debris and rocket body species were populated from the Space Environment Pathway database [3, 13]. A run with an initial grid search of revenue was conducted to gain a rough initial estimates for the solver, then a least squares optimiser determined the best fitting estimates for a_i, with b_i then determined by Equation S14. The final intercept and slope coefficients for demand are presented in Table 3.

Supplementary Note 1: Species-level effects of the bond

Figure **??** presents the same results as Figure 3 4 in the main text, disaggregated by species: S denotes constellation satellites, S_{u} non-constellation satellites, and S_{ns} CubeSats. For the larger satellites (S and S_{u}), as the bond increases, the number of compliant derelicts rises under both the 5- and 25-year PMD rules, while the number of non-compliant derelicts falls. The reduction in non-compliance exhibits diminishing returns, with a clear plateau beyond a bond level of roughly $1 million.

By contrast, the smallest class (S_{ns}) shows a different pattern: at high bond levels, the total number of CubeSats launched declines. This reflects limited annual revenue in that segment, which becomes insufficient to cover operating costs once the bond is added. As launches fall, the number of compliant derelicts also declines mechanically because there are fewer active satellites reaching end-of-life.

Supplementary Note 2: PMD compliance curve

Figure 6 visualizes the equation used in this work to estimate PMD compliance as a function of the level of compliance effort (which is assumed to be proportional to bond amount). This curve is based on the judgment of the authors, informed by informal interviews with relevant subject matter experts. As a baseline with zero bond effort, the curve begins at 45% compliance, reflecting current observed values for satellites with manouevering capability in non-naturally compliant orbits. Interviewees indicated that 80-90% large constellation PMD compliance is achievable with modest effort. Accordingly, we calibrate a 50 percent level of effort to approximately 87% compliance. Large constellation

[1] In OPUS, operational satellites will avoid collision with any other active satellite, therefore there we assume collision risk between pairs of active satellites and can be removed from this simulation.

operators generally believe that PMD compliance of 95-97% is achievable. Accordingly, we model compliance as asymptotically approaching 97%, noting that additional compliance becomes increasingly expensive with diminishing returns for very high compliance rates. Precise values for 75 and 100 percent effort are chosen to produce a smooth curve, with 100 percent effort corresponding to a bond value equal to the critical bond level b_h^* from Equation 6 in the main text, producing approximately 95% compliance.

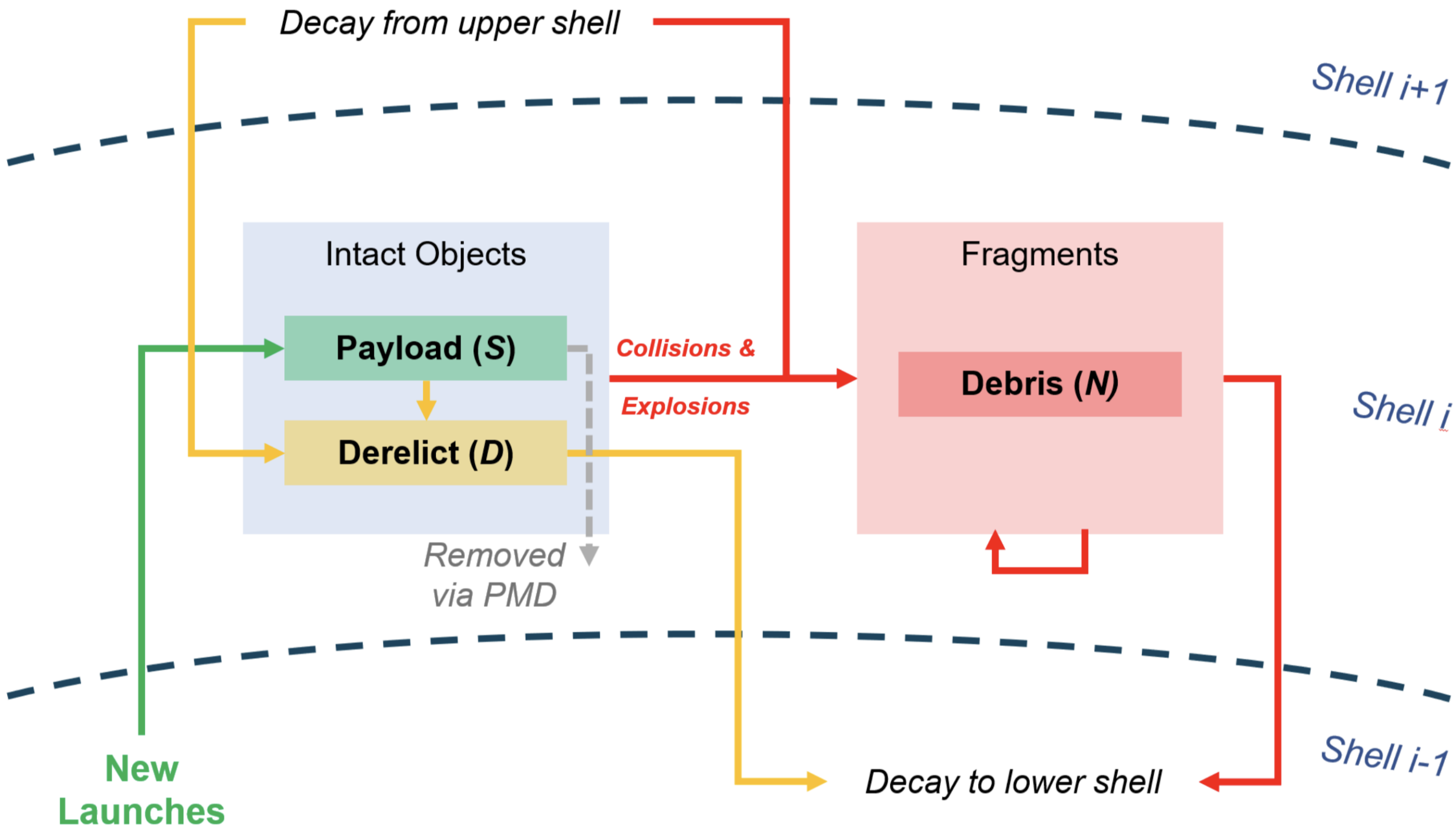

Supplementary Figure 1: MOCAT-SSEM model schematic. Qualitative representation of the source-sink evolutionary model. This is a conceptual schematic; sample size, error bars, statistical tests and exact p-values are not applicable. All symbols are defined in Supplementary Methods 1.

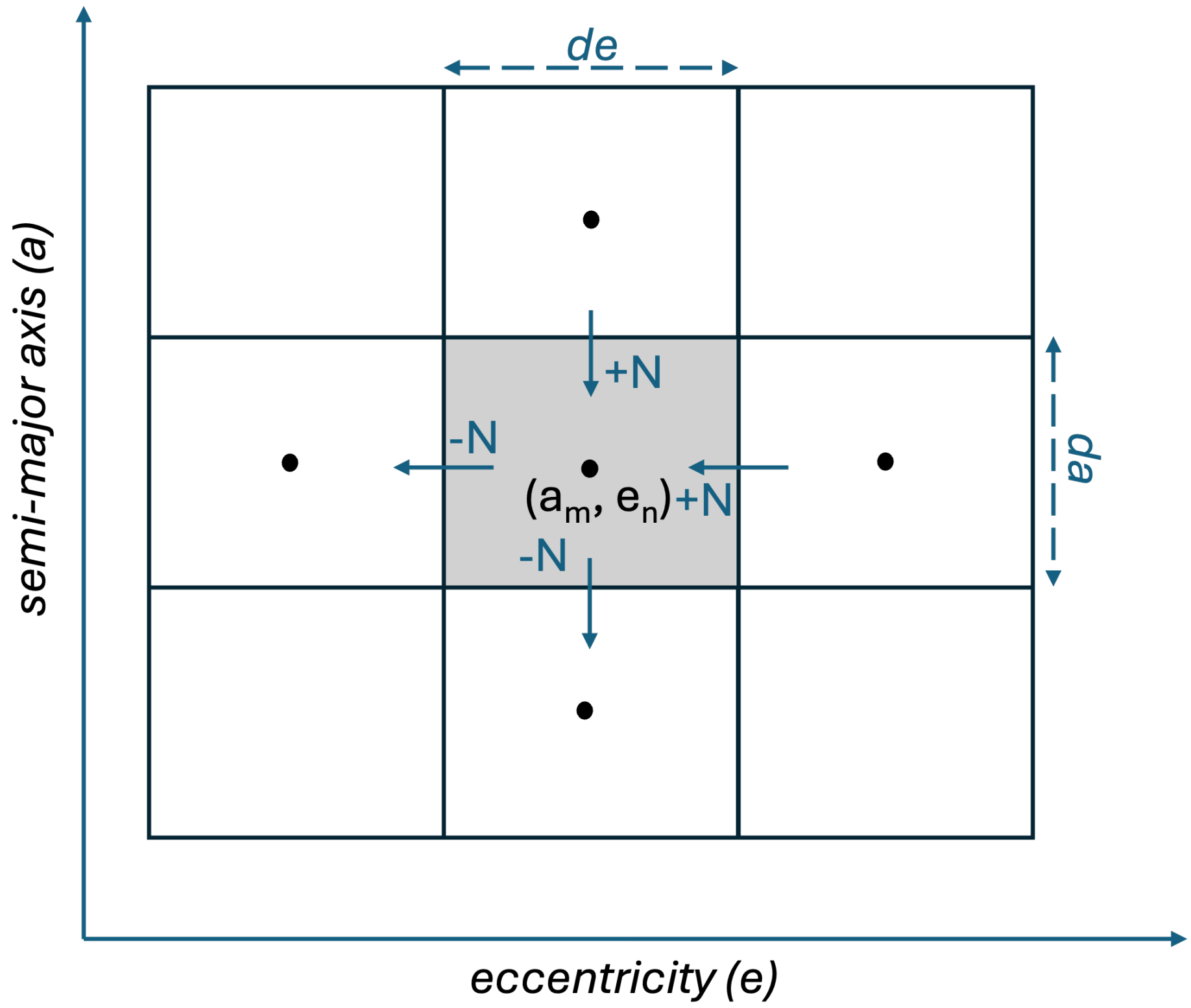

Supplementary Figure 2: Flux-model methodology in semi-major-axis and eccentricity space. The population at (a_m, e_n) increases through flux from adjacent cells at higher a and e and decreases through flux towards cells at lower a and e. The directions, symbols and cell boundaries are defined in the artwork and Supplementary Methods 1.

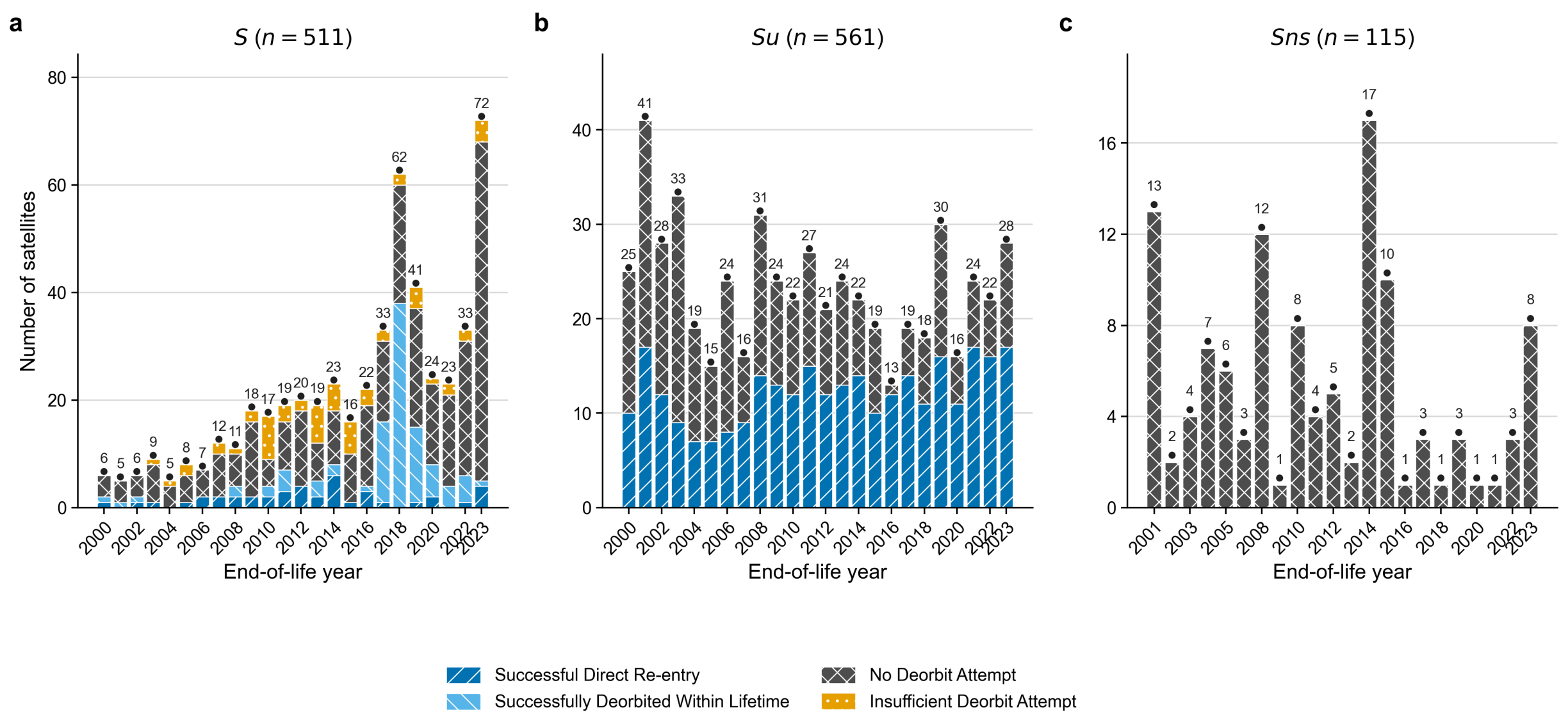

Supplementary Figure 3: Observed post-mission disposal outcomes for non-naturally compliant satellites under a 25-year lifetime criterion. Satellites are divided into the S, S_{u} and S_{ns} species. The underlying dataset contains $n = 1{,}187$ satellites: 511 constellation satellites, 561 non-constellation satellites and 115 satellites below 20 kg. Bars represent categorical counts or proportions from the complete analysed dataset rather than means derived from replicated samples. Colours and categories are defined in the figure legend. Data from [8].

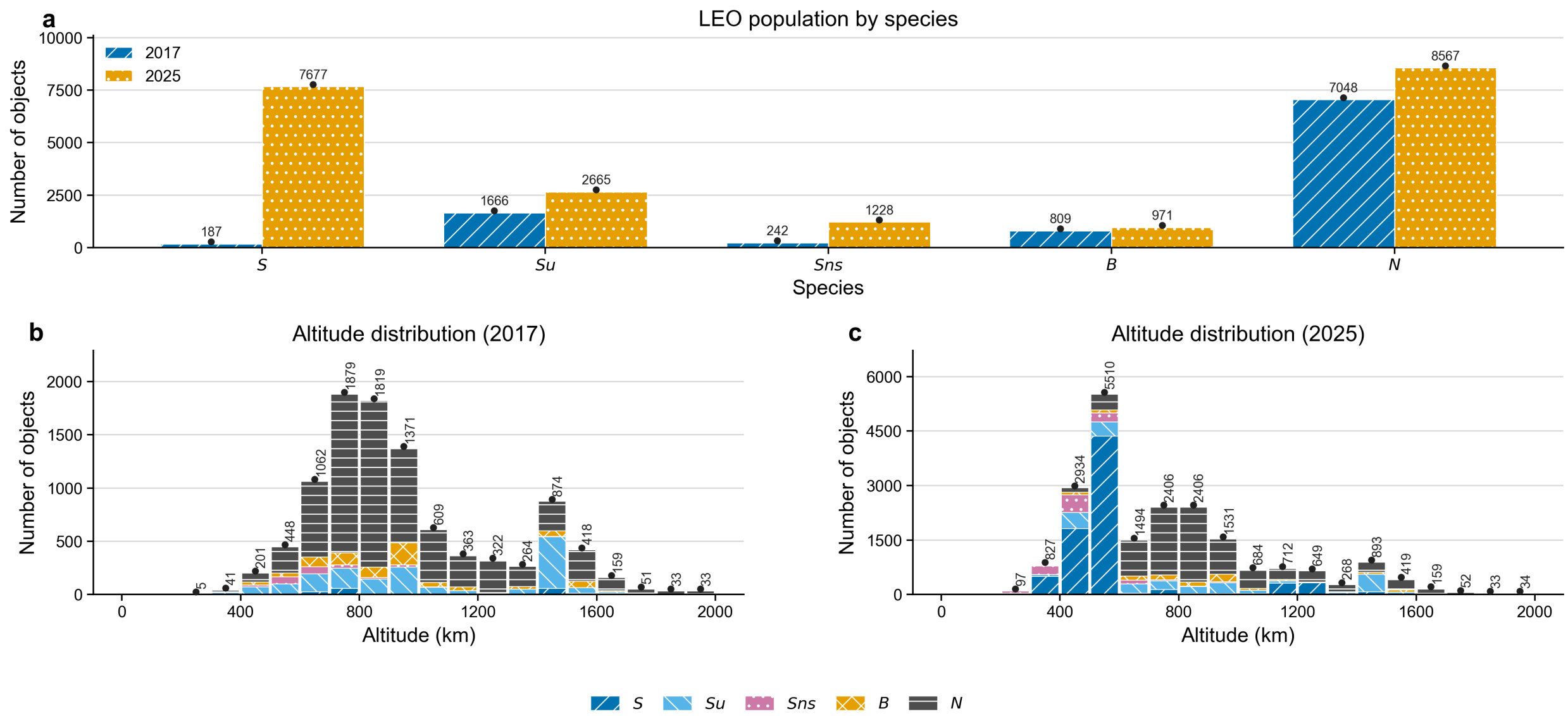

Supplementary Figure 4: Comparison of the 2017 and 2025 active-satellite populations by species. The complete analysed datasets contain $n = 45$ satellites in 2017 and $n = 11{,}570$ satellites in 2025. Bars represent complete population counts rather than means derived from replicated samples; overlaid individual data points, error bars and inferential statistical tests are not applicable. Species and colours are defined in the figure legend. Data source: Space-Track.org, augmented as described in Supplementary Methods 3.

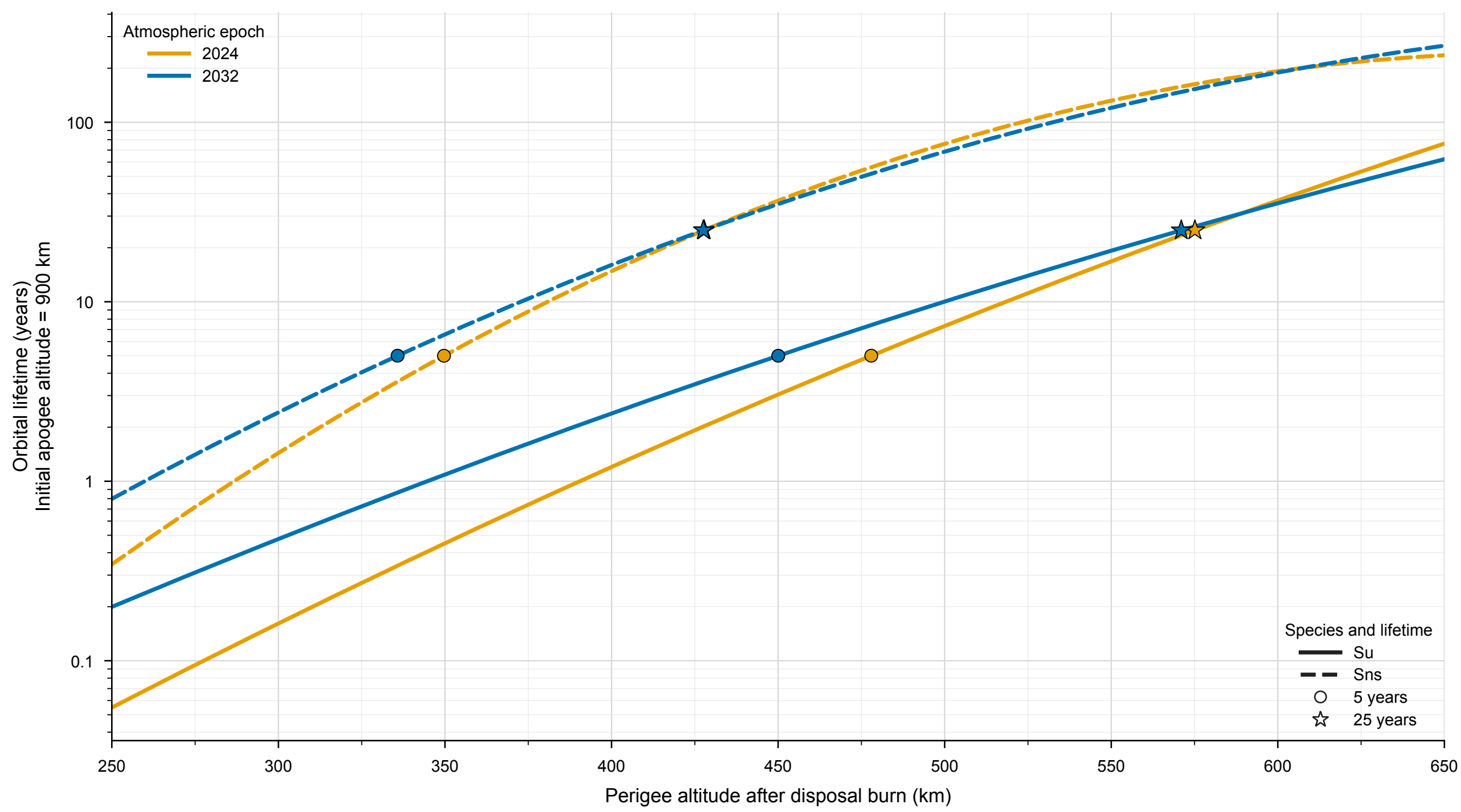

Supplementary Figure 5: Orbital lifetime of S_{u} (area-to-mass ratio 0.013 $\mathrm{m^2\,kg^{-1}}$) and S_{ns} (0.0017 $\mathrm{m^2\,kg^{-1}}$), assuming $C_{\mathrm{d}} = 2.2$ and an initial circular orbit at 900 km. Disposal starts in 2025, near solar maximum, or 2034, near solar minimum. The axes report disposal-orbit perigee altitude and orbital lifetime; reference lines identify the 5-year and 25-year compliance thresholds.

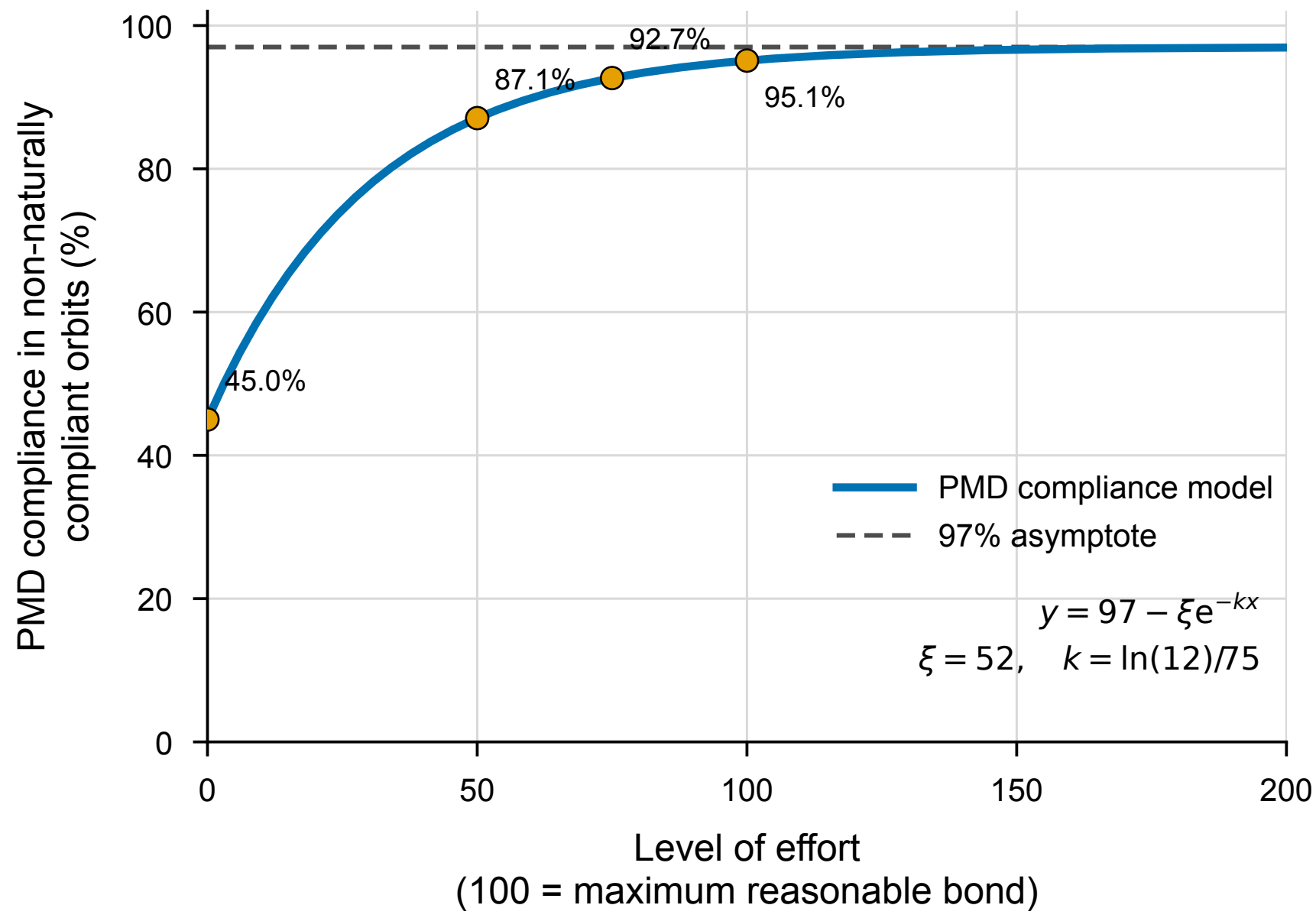

Supplementary Figure 6: Relationship between post-mission disposal effort and achievable compliance. The axes report the assumed compliance effort and the resulting compliance rate; the curve is the calibrated deterministic compliance function described in Supplementary Note 2.

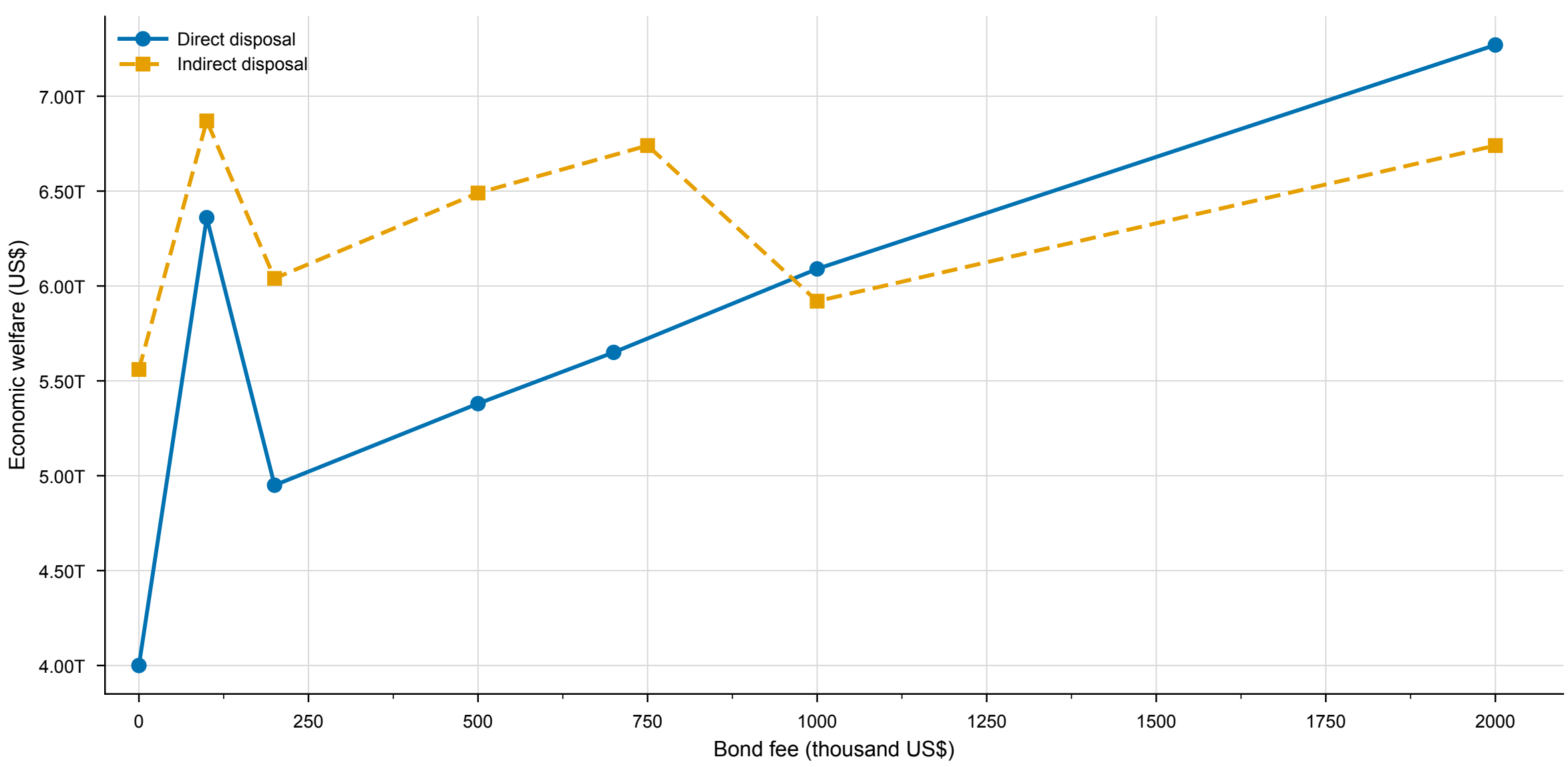

Supplementary Figure 7: Economic welfare after 25 years under PMD bonds refunded following indirect or direct disposal. The axes report bond value and final economic welfare, and line styles and symbols distinguish the two refund criteria.

MOCAT-SSEM Parameters						
**Parameter**	**Description**	S	S_u	S_{ns}	N **(Debris)**	B **(RB)**
Mass [kg]	Mass of body	521	700	20	{0.0369, 0.567, 3}	910
Radius [m]	Mean radius	2.5798	1.7186	0.1056	{0.03, 0.1321, 0.5}	1.45
Area A [m^2]	Cross-sectional area	20.9080	9.2789	0.035	{0.0108, 0.035, 0.035}	6.6122
C_d	Drag coefficient	2.2	2.2	2.2	2.2	2.2
Active	Acts as satellite	true	true	true	false	false
Manoeuvrable	Can avoid collisions	true	true	true	false	false
Slotted	Uses slotting scheme	true	false	false	false	false
Slotting effectiveness	Efficacy of slotting	1	0	0	N/A	N/A
Drag_effected	Atmospheric drag applied	false	false	false	true	true
Δt [yrs]	Operational lifetime	5	8	5	N/A	N/A
P_M	Baseline PMD success	0.95	0.65	0.65	N/A	N/A
bstar	Drag ballistic term	8.81×10^{-8}	2.35×10^{-8}	1.28×10^{-8}	{4.10e-07, 1.88e-08, 1.88e-08}	1.60×10^{-8}
RBflag	Rocket body indicator	0	0	0	0	1
**OPUS Economic Parameters**						
Sat Lifetime [yrs]	Lifetime in OPUS	5	5	5	–	–
Discount Rate	Interest rate	0.05	0.05	0.05	–	–
Revenue Intercept	α_1 (no competition)	1,665,764	2,205,401	55,701	–	–
Revenue slope	α_2	111.89	423.46	22.67	–	–
Lift price [$/kg]	Access-to-orbit cost	5000	5000	5000	–	–
Delta-v cost [$/m/s]	Propulsion cost	1000	1000	1000	–	–
Controlled PMD frac	Fraction of direct re-entries	0.6	0	0	–	–
Uncontrolled PMD frac	Derelict decay PMD	0	0.6	0.6	–	–
No-attempt PMD frac	Failed compliance	0.4	0.4	0.4	–	–

Supplementary Table 1: MOCAT-SSEM and OPUS configuration parameters. Full configuration parameters for all species used in MOCAT-SSEM and OPUS.

**Disposal Outcome**	S		S_u		S_{ns}	
	**2000–2024**	**2010–2024**	**2000–2024**	**2010–2024**	**2000–2024**	**2010–2024**
Successful Direct Re-entry (%)	8.2 (42/511)	7.1 (30/424)	52.8 (296/561)	62.3 (190/305)	0.0 (0/115)	0.0 (0/67)
Deorbited Within Lifetime (%)	19.6 (100/511)	22.4 (95/424)	0.0 (0/561)	0.0 (0/305)	0.0 (0/115)	0.0 (0/67)
No Deorbit Attempt (%)	60.5 (309/511)	58.5 (248/424)	47.2 (265/561)	37.7 (115/305)	100.0 (115/115)	100.0 (67/67)
Insufficient Attempt (%)	11.7 (60/511)	12.0 (51/424)	0.0 (0/561)	0.0 (0/305)	0.0 (0/115)	0.0 (0/67)

Supplementary Table 2: Observed post-mission disposal outcomes by satellite species. Outcomes are reported for S, S_u and S_{ns} across 2000–2024 and 2010–2024. Percentages are calculated from the precise counts shown in parentheses.

**Species**	**Intercept ($)** a	**Slope Coef.** b	**Satellites as of 1st Jan 2025**
S	1,665,764	111.89	7,677
S_u	2,205,401	423.46	2,665
S_{ns}	55,701	22.67	1,228

Supplementary Table 3: Calibrated revenue parameters and satellite populations. Final calibrated revenue intercepts (R_i) and corresponding simulated satellite populations (N_i) using Jacobian least-squares.

Bond Amount ($M)	Revenue 5-year ($B)	Revenue 25-year ($B)
$0	0.00	0.00
$100k	1.45	1.47
$200k	2.49	2.06
$500k	2.75	3.05
$700k	2.39	2.56
$1.0M	1.98	2.23
$2.0M	1.87	1.91

Supplementary Table 4: Cumulative non-compliance revenue. Cumulative revenue, in billions of US dollars, retained from non-compliant derelict fees under 5-year and 25-year PMD disposal rules.

Supplementary References

[1] Miles Lifson et al. "MOCAT-SSEM A Source-Sink Evolutionary Model for Space Debris Environment Evolutionary Modeling". In: *9th Annual Space Traffic Management Conference*. Austin, TX: IAA, 2023.

[2] N. L. Johnson et al. "NASA's new breakup model of EVOLVE 4.0". In: *Advances in Space Research* 28.9 (2001), pp. 1377–1384. DOI: 10.1016/S0273-1177(01)00423-9.

[3] Miles Lifson, Daniel Jang, and Richard Linares. "Space Environmental Governance and Decision Support using Source-Sink Evolutionary Environmental Models". In: *Advanced Maui Optical and Space Surveillance Technologies (AMOS) Conference*. Maui, 2023.

[4] Vladimir Martinusi, Lamberto Dell'Elce, and Gaëtan Kerschen. "Analytic propagation of near-circular satellite orbits in the atmosphere of an oblate planet". In: *Celestial Mechanics and Dynamical Astronomy* 123.1 (Sept. 2015), pp. 85–103. DOI: 10.1007/s10569-015-9630-7.

[5] Daniel Jang et al. "New Monte Carlo Model for the Space Environment". In: *Journal of Spacecraft and Rockets* (Mar. 2025), pp. 1–22. DOI: 10.2514/1.A36137.

[6] Bruce Bowman et al. "A New Empirical Thermospheric Density Model JB2008 Using New Solar and Geomagnetic Indices". In: *AIAA/AAS Astrodynamics Specialist Conference and Exhibit*. Reston, Virigina: American Institute of Aeronautics and Astronautics, Aug. 2008. DOI: 10.2514/6.2008-6438.

[7] Indigo Brownhall et al. "A Flexible and Improved Source Sink Evolutionary Model to Support Transdisciplinary Orbital Capacity Analysis". In: *AAS/AIAA Astrodynamics Specialist Conference*. Boston, 2025.

[8] European Space Agency. *ESA'S 2025 Annual Space Environment Report*. Tech. rep. 1. European Space Agency, 2025. URL: https://www.sdo.esoc.esa.int/environment_report/Space_Environment_Report_latest.pdf (visited on 10/21/2025).

[9] Julien Guyot and Sébastien Rouillon. "Sustainable management of space activity in low Earth orbit". In: *Journal of Environmental Economics and Policy* 13.2 (2024), pp. 188–212. DOI: 10.1080/21606544.2023.2231402.

[10] Akhil Rao, Matthew G. Burgess, and Daniel Kaffine. "Orbital-use fees could more than quadruple the value of the space industry". en. In: *Proceedings of the National Academy of Sciences* 117.23 (June 2020), pp. 12756–12762. DOI: 10.1073/pnas.1921260117. URL: https://pnas.org/doi/full/10.1073/pnas.1921260117 (visited on 01/30/2026).

[11] Frank A. Wolak. "The Welfare Impacts of Competitive Telecommunications Supply: A Household-Level Analysis". In: *Brookings Papers on Economic Activity: Microeconomics* (1996), pp. 269–340. URL: https://www.brookings.edu/wp-content/uploads/1996/01/1996_bpeamicro_wolak.pdf (visited on 02/03/2026).

[12] UCS. *Satellite Database | Union of Concerned Scientists (UCS)*. 2023. URL: https://www.ucs.org/resources/satellite-database.

[13] Miles Lifson et al. "Development of Reference Scenarios and Supporting Inputs for Space Environment Modeling". In: *Advanced Maui Optical and Space Surveillance Technologies (AMOS) Conference*. Maui, HI: Maui Economic Development Board, Sept. 2024.
