# Supplementary material for: Bonds improve post mission disposal compliance and the sustainability of low Earth orbit
Source: Nat Commun. 2026 Sep 15;17:10836. doi: 10.1038/s41467-026-77660-4 (PMC13672303; doi:10.1038/s41467-026-77660-4)
Supplement: Supplementary file 2 [file 41467_2026_77660_MOESM2_ESM.pdf]

nature portfolio

Corresponding author(s): Indigo Brownhall

Last updated by author(s): Jul 28, 2026

Reporting Summary

Nature Portfolio wishes to improve the reproducibility of the work that we publish. This form provides structure for consistency and transparency in reporting. For further information on Nature Portfolio policies, see our Editorial Policies and the Editorial Policy Checklist.

Statistics

For all statistical analyses, confirm that the following items are present in the figure legend, table legend, main text, or Methods section.

n/a	Confirmed	
☐	☒	The exact sample size (*n*) for each experimental group/condition, given as a discrete number and unit of measurement
☒	☐	A statement on whether measurements were taken from distinct samples or whether the same sample was measured repeatedly
☒	☐	The statistical test(s) used AND whether they are one- or two-sided<br>*Only common tests should be described solely by name; describe more complex techniques in the Methods section.*
☒	☐	A description of all covariates tested
☒	☐	A description of any assumptions or corrections, such as tests of normality and adjustment for multiple comparisons
☐	☒	A full description of the statistical parameters including central tendency (e.g. means) or other basic estimates (e.g. regression coefficient) AND variation (e.g. standard deviation) or associated estimates of uncertainty (e.g. confidence intervals)
☒	☐	For null hypothesis testing, the test statistic (e.g. *F*, *t*, *r*) with confidence intervals, effect sizes, degrees of freedom and *P* value noted<br>*Give P values as exact values whenever suitable.*
☒	☐	For Bayesian analysis, information on the choice of priors and Markov chain Monte Carlo settings
☒	☐	For hierarchical and complex designs, identification of the appropriate level for tests and full reporting of outcomes
☒	☐	Estimates of effect sizes (e.g. Cohen's *d*, Pearson's *r*), indicating how they were calculated

Our web collection on statistics for biologists contains articles on many of the points above.

Software and code

Policy information about availability of computer code

Data collection	The initial orbital population was retrieved from Space-Track.org using Python 3, requests (1). Custom Python scripts were used to download, filter and preprocess all catalogued objects meeting the predefined low Earth orbit inclusion criteria. The data-retrieval and preprocessing scripts are openly available in the archived code repository at Zenodo: https://doi.org/10.5281/zenodo.20506352.<br><br>Access to Space-Track requires user registration, and the original catalogue records are therefore not redistributed. The data has been manipulated to the x0.csv. The archived scripts, retrieval date, query parameters and filtering criteria allow registered users to reconstruct the initial population.
Data analysis	All modelling, simulation and analysis code used in this study is openly available from Zenodo: https://doi.org/10.5281/zenodo.20506352. The analysis was conducted using Python 3, OPUS (commit hash = c0363006e6fb3c013bae383f82a912b291e1d070) and pyssem 0.1.0.<br><br>The principal Python packages used were NumPy 1.2, pandas 2.0, SciPy 1.10, SymPy 1.11, Matplotlib. Exact software dependencies and model configurations are provided in the archived environment and requirements files.

For manuscripts utilizing custom algorithms or software that are central to the research but not yet described in published literature, software must be made available to editors and reviewers. We strongly encourage code deposition in a community repository (e.g. GitHub). See the Nature Portfolio guidelines for submitting code & software for further information.

Data

Policy information about availability of data

All manuscripts must include a data availability statement. This statement should provide the following information, where applicable:

- Accession codes, unique identifiers, or web links for publicly available datasets
- A description of any restrictions on data availability
- For clinical datasets or third party data, please ensure that the statement adheres to our policy

The datasets generated by the survey research during and/or analyzed during the current study are available in the Zenodo repository, https://doi.org/10.5281/zenodo.20506352

Research involving human participants, their data, or biological material

Policy information about studies with human participants or human data. See also policy information about sex, gender (identity/presentation), and sexual orientation and race, ethnicity and racism.

Reporting on sex and gender	There was no reporting on sex or gender within the study.
Reporting on race, ethnicity, or other socially relevant groupings	There was no reporting on race, ethnicity or other socially relevant groupings.
Population characteristics	There was no reporting on population characteristics in the study.
Recruitment	There was no recruitment of participants.
Ethics oversight	No ethical oversight was required for the study.

Note that full information on the approval of the study protocol must also be provided in the manuscript.

Field-specific reporting

Please select the one below that is the best fit for your research. If you are not sure, read the appropriate sections before making your selection.

☐ Life sciences ☐ Behavioural & social sciences ☒ Ecological, evolutionary & environmental sciences

For a reference copy of the document with all sections, see nature.com/documents/nr-reporting-summary-flat.pdf

Ecological, evolutionary & environmental sciences study design

All studies must disclose on these points even when the disclosure is negative.

Study description	This was a deterministic computational modelling study assessing the effects of refundable post-mission disposal bonds on satellite-operator behaviour and the long-term evolution of the low Earth orbit environment. The OPUS economic model was coupled with MOCAT-SSEM to simulate interactions between operator decisions and the orbital environment over a 25-year period. Scenarios systematically varied predefined policy and market parameters, including bond value, post-mission disposal requirement, refund mechanism and market coverage. No field or laboratory experiments were undertaken.
Research sample	The empirical input consisted of all catalogued space objects meeting the predefined low Earth orbit inclusion criteria at the model initialisation epoch in 2025. Orbital catalogue records were downloaded from Space-Track.org and used to initialise the model population across the relevant object classes and orbital bins. The final initial population contained n = 21,209 catalogued objects. No subset of eligible objects was sampled.
Sampling strategy	No probabilistic sampling strategy was used. The initial condition was constructed as a census of all objects in the Space-Track catalogue that met the predefined low Earth orbit inclusion criteria at the selected epoch. The sample size was therefore determined by the number of eligible catalogued objects rather than by statistical power calculations. No a priori sample-size calculation was performed because the study was a deterministic computational scenario analysis and did not estimate effects from a sampled population.
Data collection	The catalogue data were downloaded by the lead author from Space-Track.org on 01/09/2025, from space-track.org and a Python preprocessing script. The records were supplied in machine-readable form and imported directly into Python. No physical instruments, field observations or manual measurements were used. The preprocessing script applied the predefined orbital inclusion criteria and assigned eligible objects to the corresponding model species and orbital bins.
Timing and spatial scale	The empirical dataset represented a single catalogue snapshot at the model initialisation epoch of 01-03-2025. It was not a longitudinal sample collected between 1967 and 2025; earlier launch dates were historical attributes of objects present in the catalogue snapshot. The spatial domain was Earth-centred low Earth orbit, covering the modelled altitude range up to 2,000 km.

Data exclusions	Objects outside the predefined low Earth orbit domain were excluded before model initialisation. No additional post hoc exclusions were made.
Reproducibility	All data preprocessing, model configuration, simulation and analysis steps were performed using scripted workflows. The model source code, configuration files, analysis scripts and model-generated data underlying the figures are archived at https://doi.org/10.5281/zenodo.20506352. The simulations are deterministic, and the archived configuration files and documented software versions allow the analyses to be reproduced.
Randomization	Randomisation was not applicable. There were no participants, sampled groups or experimental units requiring random allocation. Policy scenarios were defined in advance and evaluated systematically using deterministic simulations.<br><br>The study did not analyse observational covariates. For each comparison, the initial orbital population and all parameters not designated as treatment factors were held constant. Only the predefined policy or market parameters under investigation were varied. Additional parameters were varied separately in the sensitivity analyses described in the manuscript.
Blinding	Blinding was not applicable because the study did not involve participants, experimental group allocation or subjective outcome assessment. Investigators were aware of the scenario parameters, but all outcomes were calculated automatically by the modelling software using predefined equations and analysis procedures.

Did the study involve field work? ☐ Yes ☒ No

Reporting for specific materials, systems and methods

We require information from authors about some types of materials, experimental systems and methods used in many studies. Here, indicate whether each material, system or method listed is relevant to your study. If you are not sure if a list item applies to your research, read the appropriate section before selecting a response.

Materials & experimental systems

n/a	Involved in the study	
☒	☐	Antibodies
☒	☐	Eukaryotic cell lines
☒	☐	Palaeontology and archaeology
☒	☐	Animals and other organisms
☒	☐	Clinical data
☒	☐	Dual use research of concern
☒	☐	Plants

Methods

n/a	Involved in the study	
☒	☐	ChIP-seq
☒	☐	Flow cytometry
☒	☐	MRI-based neuroimaging

Plants

Seed stocks	*Report on the source of all seed stocks or other plant material used. If applicable, state the seed stock centre and catalogue number. If plant specimens were collected from the field, describe the collection location, date and sampling procedures.*
Novel plant genotypes	*Describe the methods by which all novel plant genotypes were produced. This includes those generated by transgenic approaches, gene editing, chemical/radiation-based mutagenesis and hybridization. For transgenic lines, describe the transformation method, the number of independent lines analyzed and the generation upon which experiments were performed. For gene-edited lines, describe the editor used, the endogenous sequence targeted for editing, the targeting guide RNA sequence (if applicable) and how the editor was applied.*
Authentication	*Describe any authentication procedures for each seed stock used or novel genotype generated. Describe any experiments used to assess the effect of a mutation and, where applicable, how potential secondary effects (e.g. second site T-DNA insertions, mosiacism, off-target gene editing) were examined.*
