# Supplementary material for: Bonds improve post mission disposal compliance and the sustainability of low Earth orbit
Source: Nat Commun. 2026 Sep 15;17:10836. doi: 10.1038/s41467-026-77660-4 (PMC13672303; doi:10.1038/s41467-026-77660-4)
Supplement: Supplementary file 3 [file 41467_2026_77660_MOESM3_ESM.pdf]

nature portfolio

Peer Review File

Bonds Improve Post Mission Disposal Compliance and the Sustainability of Low Earth Orbit

Corresponding Author: Mr Indigo Brownhall

This file contains all reviewer reports in order by version, followed by all author rebuttals in order by version.

Version 0:

Reviewer comments:

Reviewer #1

(Remarks to the Author)
The paper studies the effect of deorbiting performance bonds as a policy tool for post-mission satellite disposal compliance. Overall, I find the study highly relevant and well executed. I recommend a few revisions to clarify the results and extend their usefulness to a broad audience (policymakers, academics, and professionals who may not have the technical training or background to fully understand the implications of the findings).

First, the economic part of the model builds on Adilov et al. (Economics Letters, 2023) theoretical foundations for using performance bonds to incentivize firms to deorbit their satellites. I found it particularly interesting that the suggested maximum revenue collection from deorbiting bonds (around $500,000 per satellite) is close to the compliance-oriented numerical example in Adilov et al.'s study (a bond amount close to $500,000 or 5% of the satellite's value). However, it wasn't clear from the paper what percentage of the satellite values different bond amounts represent. I suggest discussing bond values as percentages of the satellite values, so that they provide a meaningful comparative evaluation for commercial satellite operators. This would not require additional estimation; rather, it would involve discussing the existing results in percentage terms, as it was not straightforward to calculate this information from the presented data and the tables.

Second, to better inform policymakers about potential trade-offs, I suggest including a single figure that illustrates the relationship between changes in the bond amount and changes in debris pollution or disposal compliance rates. Such a visualization could offer intuitive cost-benefit guidance for policymakers.

Third, I recommend rewriting the introduction section to make it more easily accessible to non-technical readers. A more accessible presentation will ensure the paper's contributions reach a wider audience.

As I noted before, this is a strong and important paper, and revising it with the feedback above would further strengthen it and flesh out its implications for policy.

(Remarks on code availability)

Reviewer #2

(Remarks to the Author)
The manuscript is very interesting and innovative in the approach proposed. The inclusion of graphs and tables helps make the analysis clearer and more accessible to the reader.

One aspect that emerged during the review concerns the possibility of presenting some of the reported data in a more precise and less approximate manner. A higher level of accuracy in the presentation of the data would further strengthen the overall quality of the work and the solidity of the arguments developed.

(Remarks on code availability)

Reviewer #3

(Remarks to the Author)
I co-reviewed this manuscript with one of the reviewers who provided the listed reports. This is part of the Nature Communications initiative to facilitate training in peer review and to provide appropriate recognition for Early Career Researchers who co-review manuscripts.

(Remarks on code availability)

Reviewer #4

(Remarks to the Author)
I co-reviewed this manuscript with one of the reviewers who provided the listed reports. This is part of the Nature Communications initiative to facilitate training in peer review and to provide appropriate recognition for Early Career Researchers who co-review manuscripts.

(Remarks on code availability)

Version 1:

Reviewer comments:

Reviewer #1

(Remarks to the Author)
The reviewers addressed my comments on the earlier draft of the paper.

(Remarks on code availability)

Reviewer #2

(Remarks to the Author)
The revisions made have significantly strengthened the overall quality of the research, which now appears solid, coherent, and able to present the selected case study in the best possible way.

(Remarks on code availability)

Reviewer #3

(Remarks to the Author)
I co-reviewed this manuscript with one of the reviewers who provided the listed reports. This is part of the Nature Communications initiative to facilitate training in peer review and to provide appropriate recognition for Early Career Researchers who co-review manuscripts.

(Remarks on code availability)
The authors have responded constructively to the points raised during the review process. The revised version is clearer, better structured, and more effective in presenting the selected case study. The manuscript now provides a solid contribution, and I do not have further substantive concerns.

Reviewer #4

(Remarks to the Author)
I co-reviewed this manuscript with one of the reviewers who provided the listed reports. This is part of the Nature Communications initiative to facilitate training in peer review and to provide appropriate recognition for Early Career Researchers who co-review manuscripts.

(Remarks on code availability)

Open Access This Peer Review File is licensed under a Creative Commons Attribution 4.0 International License, which permits use, sharing, adaptation, distribution and reproduction in any medium or format, as long as you give appropriate credit to the original author(s) and the source, provide a link to the Creative Commons license, and indicate if changes were made.
In cases where reviewers are anonymous, credit should be given to 'Anonymous Referee' and the source.
The images or other third party material in this Peer Review File are included in the article's Creative Commons license, unless indicated otherwise in a credit line to the material. If material is not included in the article's Creative Commons license and your intended use is not permitted by statutory regulation or exceeds the permitted use, you will need to obtain permission directly from the copyright holder.
To view a copy of this license, visit https://creativecommons.org/licenses/by/4.0/

Reviewer #1

Comment 1. *The paper studies the effect of deorbiting performance bonds as a policy tool for post-mission satellite disposal compliance. Overall, I find the study highly relevant and well executed.*

Response. We thank the reviewer for their positive assessment of the manuscript and for recognising the relevance and execution of the study. We are grateful for the constructive comments that follow, which have helped us improve the clarity, accessibility, and policy relevance of the paper. We have addressed each point in detail below.

Comment 2. *I recommend a few revisions to clarify the results and extend their usefulness to a broad audience (policymakers, academics, and professionals who may not have the technical training or background to fully understand the implications of the findings).*

First, the economic part of the model builds on Adilov et al. (Economics Letters, 2023) theoretical foundations for using performance bonds to incentivize firms to deorbit their satellites. I found it particularly interesting that the suggested maximum revenue collection from deorbiting bonds (around $500,000 per satellite) is close to the compliance-oriented numerical example in Adilov et al.'s study (a bond amount close to $500,000 or 5% of the satellite's value). However, it wasn't clear from the paper what percentage of the satellite values different bond amounts represent. I suggest discussing bond values as percentages of the satellite values, so that they provide a meaningful comparative evaluation for commercial satellite operators. This would not require additional estimation; rather, it would involve discussing the existing results in percentage terms, as it was not straightforward to calculate this information from the presented data and the tables.

Response. We thank the reviewer for this helpful suggestion. You are correct that the previous version did not include enough information to back-out the percentage value, and we agree that expressing the bond values as percentages of representative satellite values provides an intuitive basis for comparison with commercial decision-making and with the benchmark discussed by Adilov et al.

We have therefore added percentage benchmarks to the manuscript in two places: the caption of Figure 2 and the first subsection of the Results. Here, a $500,000 bond corresponds to an average value of a satellite of approximately 17%. This is higher than the 5% illustrative value discussed by Adilov et al., but remains of the same order of magnitude for the larger satellite classes.

Comment 3. *Second, to better inform policymakers about potential trade-offs, I suggest including a single figure that illustrates the relationship between changes in the bond amount and changes in debris pollution or disposal compliance rates. Such a visualization could offer intuitive cost-benefit guidance for policymakers.*

Response. We thank the reviewer for this suggestion. We agree that a direct visual comparison between bond value and environmental response provides clearer guidance for policymakers.

In response, we have extended Figure 2 to include an additional panel showing how the number of derelict satellites responds to changes in the bond amount. This panel distinguishes between compliant and non-compliant derelicts, thereby illustrating how increasing the bond value affects the population of objects contributing to debris pollution. The revised Figure 2 now presents these results alongside the UMPY response. The left-hand panel therefore shows the direct pollution response, measured through the derelict population, while the right-hand panel shows the wider environmental impact of those derelicts on the orbital environment through the UMPY metric.

Together, these panels provide a clearer cost-benefit interpretation of the bond scenarios by showing both the policy mechanism and the impact on the environment.

Comment 4. *Third, I recommend rewriting the introduction section to make it more easily accessible to non-technical readers. A more accessible presentation will ensure the paper's contributions reach a wider audience.*

Response. We thank the reviewer for this positive assessment of the manuscript and for recognising the relevance of the study. We agree that the introduction should be presented in a way that is accessible to a wider audience. In response, we have revised the introduction, by removing unnecessary acronyms, jargon, and detail and moving them to more appropriate parts of the manuscript.

Comment 5. *As I noted before, this is a strong and important paper, and revising it with the feedback above would further strengthen it and flesh out its implications for policy.*

Response. We thank the reviewer for their encouraging assessment of the manuscript and for recognising its potential contribution to policy discussions on post-mission disposal and space sustainability.

Reviewer #2

Comment 6. *The manuscript is very interesting and innovative in the approach proposed. The inclusion of graphs and tables helps make the analysis clearer and more accessible to the reader.*

One aspect that emerged during the review concerns the possibility of presenting some of the reported data in a more precise and less approximate manner. A higher level of accuracy in the presentation of the data would further strengthen the overall quality of the work and the solidity of the arguments developed.

Response. We thank the reviewer for their positive assessment of the manuscript and for recognising the value of the figures and tables in supporting the analysis. We also appreciate the reviewer's careful attention to the precision with which the results are presented.

In response, we have revised the manuscript to improve the accuracy and clarity of the reported

data. In particular, we are grateful for the reviewer's identification of the incorrect estimate in the ESA future-collision figure, as well as several grammatical and presentation issues. These have now been corrected in the revised manuscript.

We also recognise that the explanation of the third and final results section could have been clearer. Figure 4 presents four subplots showing how UMPY and economic welfare vary with both bond value and market leakage. The distinction between the left- and right-hand columns refers to the share of the market to which the bond is applied. Specifically, the left-hand column corresponds to a bond applied to 20% of the market; this value is not a rounded result from the plotted data. We have revised the figure caption and accompanying text to make this interpretation explicit.

We believe these changes improve the precision of the data presentation and strengthen the clarity of the policy interpretation.

Reviewer #3 & #4

Comment 1. *I co-reviewed this manuscript with one of the reviewers who provided the listed reports. This is part of the Nature Communications initiative to facilitate training in peer review and to provide appropriate recognition for Early Career Researchers who co-review manuscripts.*

Response. We thank the co-reviewer for their contribution to the review process and for their time in assessing the manuscript.
